# Read Length Dominates Phylogenetic Placement Accuracy of Ancient DNA Reads

**DOI:** 10.1093/molbev/msaf006

**Published:** 2025-01-17

**Authors:** Ben Bettisworth, Nikolaos Psonis, Nikos Poulakakis, Pavlos Pavlidis, Alexandros Stamatakis

**Affiliations:** Institute of Computer Science, Foundation for Research and Technology-Hellas (FORTH), Heraklion, Greece; Ancient DNA Lab, Institute of Molecular Biology and Biotechnology (IMBB), Foundation for Research and Technology-Hellas (FORTH), Heraklion, Greece; Ancient DNA Lab, Institute of Molecular Biology and Biotechnology (IMBB), Foundation for Research and Technology-Hellas (FORTH), Heraklion, Greece; Natural History Museum of Crete, School of Sciences and Engineering, University of Crete, Heraklion, Greece; Department of Biology, School of Sciences and Engineering, University of Crete, Heraklion, Greece; Institute of Computer Science, Foundation for Research and Technology-Hellas (FORTH), Heraklion, Greece; Department of Biology, School of Sciences and Engineering, University of Crete, Heraklion, Greece; Institute of Computer Science, Foundation for Research and Technology-Hellas (FORTH), Heraklion, Greece; Computational Molecular Evolution Group, Heidelberg Institute for Theoretical Studies, Heidelberg, Germany; Institute for Theoretical Informatics, Karlsruhe Institute of Technology, Karlsruhe, Germany

**Keywords:** ancient DNA, phylogenetic placement, alignment

## Abstract

A common problem when analyzing ancient DNA data is to identify the species that corresponds to the recovered analyzing ancient DNA sequence(s). The standard approach is to deploy sequence similarity-based tools, such as BLAST. However, as analyzing ancient DNA reads may frequently stem from unsampled taxa due to extinction, it is likely that there is no exact match in any database. As a consequence, these tools may not be able to accurately place such reads in a phylogenetic context. Phylogenetic placement is a technique where a read is placed onto a specific branch of a phylogenetic reference tree, which allows for a substantially finer resolution when identifying reads. Prior applications of phylogenetic placement have deployed only on data from extant sources. Therefore, it is unclear how the analyzing ancient DNA damage affects phylogenetic placement’s applicability to analyzing ancient DNA data. To investigate how analyzing ancient DNA damage affects placement accuracy, we re-implemented a statistical model of analyzing ancient DNA damage. We deploy this model, along with a modified version of the existing assessment pipeline PEWO, to 7 empirical datasets with 4 leading tools: APPLES, EPA-Ng, pplacer, and RAPPAS. We explore the analyzing ancient DNA damage parameter space via a grid search in order to identify the analyzing ancient DNA damage factors that exhibit the largest impact on placement accuracy. We find that the frequency of DNA backbone nicks (and consequently read length) has the, by far, largest impact on analyzing ancient DNA read placement accuracy, and that other factors, such as misincorporations, have a negligible effect on overall placement accuracy.

## Introduction

The analysis of DNA obtained from archaeological sites, as well as paleontological and sediment samples has changed our understanding of life in the past (Haile et al. 2009; Pedersen et al. 2015; Der Sarkissian et al. 2017). However, the characteristics of ancient DNA (aDNA) differ substantially from those of contemporary samples. Due to several degradation processes (including deamination and fragmentation), analyzing aDNA data exhibit more challenges than analyzing DNA obtained from contemporary sources. The DNA repair mechanisms, which guarantee that DNA in the cells is not damaged, become inactive when an organism dies, which allows DNA to accumulate damage. This *postmortem* damage accumulation process often results in poor quality of the recovered genetic material from ancient and historical samples. This is to say, analyses using aDNA samples are likely to yield incorrect results, due to both the short length and accumulated damage (Kivisild 2017).

The DNA damage accumulation rate depends on time, temperature, acidity, and other environmental factors (Sawyer et al. 2012), which complicate the prediction of the *postmortem* damage level for a given sample. Nonetheless, the damage itself can be described via a straight-forward model, originally proposed by Briggs et al. (2007). Of the possible damage types that DNA accumulates as it ages, the Briggs model of aDNA damage (hereafter, simply the Briggs model) incorporates 2 major types: nicks to the DNA backbone, which shred the DNA into shorter fragments; and point errors caused by cytosine (C) deamination. When cytosine is deaminated it transforms into uracil (U) and is subsequently read as thymine (T) instead of cytosine (C) during DNA sequencing. This process is commonly known as C to T damage, which we denote as C→T. This damage is observed more frequently toward the 5′ end of the ancient sequence reads of double-stranded libraries, as well as toward both the 5′ and 3′ ends of single-stranded library reads. There exists an analogous G to A damage (which we denote as G→A) that is predominantly observed at the 3′ read ends of double-stranded libraries. This is induced by the genomic library preparation procedure in the wet lab due to DNA complementarity.

Under the Briggs model, the deamination rate is controlled by the location of the respective base in relation to an overhang. An overhang is a section of the sequence where the complementary strand has been separated. The bases of the primary strand have, therefore, been exposed to the environment without the stabilizing presence of the complementary strand. These overhangs produce so-called single-stranded regions (in contrast to double-stranded regions), which exhibit substantially higher rates of deamination due to their unprotected exposure to environmental conditions. For example, Briggs et al. found deamination rates in the single-stranded region to be an order of magnitude higher than in the double-stranded region. As overhangs necessarily occur at strand ends, the vast majority of C to T damage (and consequently also G to A damage) occurs at the beginning and the end of a read. For an example read of 100 bp, the overhang only needs to be ≈3 bases long to exhibit more expected errors than the double-stranded region. Many reads are substantially shorter, often only comprising 30 bp due to filtering steps after the sequencing process. Hence, due to the short length with respect to the overhang size, most damage occurs at read ends. This induces a characteristic “U” shaped curve of misincorporated point differences as shown in Briggs et al. (2007) and Sawyer et al. (2012).

One major challenge in aDNA data analysis is taxonomic classification, that is, assigning a DNA read to a taxon. In addition to the processes described above, aDNA samples are also likely to be contaminated by exogenous sources. Determining the organism a read originally belonged to allows researchers to make inferences based on the read context, for example by refining the range, both spatial and temporal, of extinct species (Haile et al. 2009) or uncovering admixture events in archaic hominins, for instance (Slatkin and Racimo 2016). However, aDNA degradation results in both, small DNA fragments, and erroneous point differences, yielding identification challenging.

Prior data analyses have attempted to assign aDNA reads via tools, such as BLAST (Haile et al. 2009) or Kraken (Wood and Salzberg 2014). These tools match a short (a)DNA query sequence or read against a (possibly large) database of known sequences. However, sequence similarity-based methods only match against known sequences, or equivalently, sequences located at the tips of a corresponding phylogenetic tree. However, if the query sequence is sampled from a heretofore unknown, extinct, or hypothetical species, a similarity-based taxonomic assignment will be imprecise. These methods will indicate a single clade and possibly a confidence associated with the assignment to that clade. Yet, if multiple clades contain a closely matching reference sequence, similarity-based methods will typically only assign the read to a single clade.

An alternative method for taxonomic identifications is to deploy phylogenetic placement, as implemented in EPA-Ng, APPLES, pplacer, or RAPPAS (Matsen et al. 2010; Barbera et al. 2019; Linard et al. 2019; Balaban et al. 2020). Phylogenetic placement methods place query sequences into a phylogenetic reference tree. Phylogenetic placement is a technique that places unknown, sampled sequences, called query sequences, into a known reference tree inferred from known/named sequences, called reference sequences. By using a phylogeny, placement is able to match query sequences against hypothetical and unsampled species (i.e. an evolutionary history), as well as against known species. Therefore, placement-based taxonomic assignment is often more specific and accurate than a mere assignment to a potentially taxon-rich clade (Berger et al. 2011). This is because phylogenetic placement will assign the query sequence to a specific branch of the phylogenetic reference tree. For a comprehensive review of phylogenetic placement methods that also includes a plethora of references to empirical data analyses on contemporary DNA data please refer to Czech et al. (2022).

For the purposes of aDNA, placement offers 2 advantages over traditional methods of taxonomic identification: First, placement can assign a query to a species that is not in the dataset by placing the query onto an internal branch; and second the results of placement are substantially more specific, since tools such as EPA-Ng and RAPPAS will also infer the phylogenetic distance between the query and the rest of the tree. However, a recent article raised the question if phylogenetic placement, which was originally devised for analyzing contemporary DNA sequences, can adequately identify aDNA sequences due to their short read length and characteristic damage (Martiniano et al. 2022). However, a systematic investigation of the effects of aDNA damage on phylogenetic placement accuracy has yet to be conducted. In the following, we therefore systematically investigate the impact of aDNA damage on placement accuracy. We focus on aDNA damage so as to isolate the effects of, aDNA damage, which is relatively novel from a bioinformatic standpoint. Evidently, other sources of error, such as contamination and sequencing error also need to be taken into account when analyzing aDNA data. However, there exist standard techniques to alleviate these errors (for an overview, please see Pedersen et al. 2015), whereas aDNA damage is an inherent source of error that is inexorably linked with the analysis of aDNA sequences.

To this end, we have modified the existing phylogenetic placement benchmark pipeline PEWO (Linard et al. 2021) by including a step for injecting aDNA damage into the query sequences. Using this modified pipeline, we conduct appropriate accuracy tests using the following placement tools: EPA-Ng (Barbera et al. 2019), APPLES (Balaban et al. 2020), pplacer (Matsen et al. 2010), and RAPPAS (Linard et al. 2019).

## Methods

To evaluate the effects of aDNA damage on phylogenetic placement accuracy, we initially re-implemented the popular aDNA damage simulator Gargammel (Renaud et al. 2017) as an open source Python program which we call PyGargammel (available at https://github.com/computations/pygargammel). This re-implementation is required to better control the particular type and magnitude of aDNA damage (specifically the parameters of the Briggs Model that is implemented in Gargammel and PyGargammel), as this is difficult in Gargammel. Specifically, in contrast to Gargammel, PyGargammel is substantially simpler, provides detailed information about the damage simulation process. This makes PyGargammel much more ideal for the purpose of benchmarking phylogenetic tools, as the information about the damage process can help inform future development of these tools. For both simulators, the input is a FASTA file containing one or more sequences. Gargammel simulates reads, and therefore outputs the results as FASTQ files, which need to subsequently be assembled *and* aligned (output as BAM) in order to be suitable for use in PEWO. However, as we intend to isolate the effects of aDNA damage exclusively on placement results, our damage simulator implementation allows to skip these potentially confounding steps. To this end, PyGargammel outputs its results as sequences in FASTA format, with an option to preserve alignment with the original, undamaged, sequence. We discuss PyGargammel in more detail in Section “Simulating Ancient DNA Damage”.

To facilitate running multiple phylogenetic placement programs on alignments with simulated damage from PyGargammel, we used the placement accuracy assessment pipeline PEWO (Linard et al. 2021). However, executing PEWO with PyGargammel, required several modifications to PEWO, which we detail in Section “Modifications to the PEWO Pipeline”.

Finally, several damage simulation parameters needed to be chosen for this application. Instead of selecting single parameters or sampling the parameter space uniformly, we chose to perform a grid search over possible damage parameter configurations. These choices are detailed in Section “Pipeline Parameters”.

### Data

We investigated the effects of aDNA damage on placement accuracy using 7 empirical datasets. The dataset properties, such as taxon number and site count, are provided in Table 1. We sought to include a wide range of datasets, including standard phylogenetic placement applications for environmental DNA (Srinivasan et al. 2012; Sunagawa et al. 2015; Mahé et al. 2017), but also datasets from prior aDNA studies. These datasets comprise for the most part larger animals, such as Aurochs (Gurke et al. 2021), Land Snails (Psonis et al. 2022a), Hippos (Psonis et al. 2022b), and Elephants (Psonis et al. 2020). These datasets therefore represent a broad spectrum of aDNA identification tasks. We chose to use 7 datasets in order to focus on parameter exploration, rather than expending a large number of resources on a shallow exploration of many datasets. Nonetheless, we have sought to include diverse enough datasets so that many typical aDNA data applications are well represented by our results.

**Table 1:** Table of empirical datasets used for experiments

Dataset	Taxa	# of Sites	GC %	Source	Reference
ds01	141	16,354	39	Aurochs	(Gurke et al. 2021)
ds02	77	932	26	Land Snails	(Psonis et al. 2022a)
ds03	36	1,784	43	Hippos	(Psonis et al. 2022b)
ds04	30	16,025	38	Elephant	(Psonis et al. 2020)
ds05	512	4,686	17	Soil	(Mahé et al. 2017)
ds06	3,748	3,374	22	Ocean	(Sunagawa et al. 2015)
ds07	797	2,763	25	Vaginal Swabs	(Srinivasan et al. 2012)

Source is a short description of either the environment the sample was collected from, or the taxonomic clade the samples represent. GC % is the average GC content for all sequences in the alignment.

In addition to the 7 empirical datasets, we also investigated the impact of GC content on placement accuracy in the presence of aDNA damage using 9 simulated datasets. Under the model, we use to simulate aDNA damage, only sites that contain a G or C are eligible to be damaged (see Section “Simulating Ancient DNA Damage”). Therefore, the quantity of misincorporations in a fragment will be proportional to the GC content of the initial source sequence. As an increasing number of misincorporations could affect placement accuracy, we investigate the effects of varying levels of GC content to ensure that the results from the empirical analysis are likely to hold for datasets with higher levels of GC content. The description of how these datasets was generated, as well as the results from these datasets are described in the Supplementary material online.

### The PEWO Pipeline

Instead of re-implementing an evaluation pipeline for analyzing phylogenetic placement accuracy, we appropriately modified the existing PEWO pipeline (Linard et al. 2021) to also support damage simulation. Therefore, we enhanced PEWO by directly integrating simulation of damaged sequences via a damage simulator we call PyGargammel. The details of PyGargammel and how it simulates damage are found in Section “Simulating Ancient DNA Damage”.

Given a multiple sequence alignment (MSA) and an associated phylogeny, PEWO performs analysis by:

**Pruning** Pruning the taxa contained in a randomly selected subtree, as well as removing and fragmenting the sequences contained in this subtree from the MSA,

**Alignment** Realigning the pruned and shortened sequences all at once with HMMER (Eddy 2011),

**Placement** Running various placement programs to place the pruned sequences back on the reference tree,

**Assessment** and Computing and summarizing the placement error with regards to the ground truth placement.

PEWO is designed to investigate the accuracy of phylogenetic placement tools using an MSA and the corresponding alignment, and, as part of the pruning process, it will fragment the pruned portion of the input MSA into multiple parts. This fragment is conducted by cutting the pruned sequences into regions of equal length, which is specified as a configuration parameter. However, the behavior of aDNA fragmentation substantially differs from sequence fragmentation as implemented in PEWO. Therefore, we implement our own fragmenting method (detailed in Section “Simulating Ancient DNA Damage”), and disable the sequence fragmentation in PEWO.

PEWO measures the performance of placement tools using 2 metrics: node distance (ND) and expected node distance (eND). In short, ND is the topological distance between the true placement and the best inferred placement position. The eND is the sum of the node distances for all reported placement positions weighted by the likelihood weight ratio of that placement. For more information about phylogenetic placement accuracy assessment please see Matsen et al. (2010) and Berger et al. (2011). For more information on the specific computation of ND and eND, please refer to the original PEWO publication (Linard et al. 2021).

#### Modifications to the PEWO Pipeline

We modified PEWO by inserting PyGargammel both before and after the alignment step. Injecting aDNA sequence damage before alignment is more realistic as aligning reads is a necessary step prior to placement for empirical sequences. However, we also inject aDNA damage *after* the alignment stage, so as to isolate the effects of aDNA damage on placement accuracy, without any confounding alignment error. Unless otherwise stated, we report placement accuracy for damage injection after alignment. We conducted exploratory experiments with damage inserted before alignment, however a thorough investigation of the joint alignment and placement error is beyond the scope of this paper. Specifically, we wish to investigate the case when alignment error is negligible to determine the baseline effectiveness of placement algorithms.

Therefore, the operation order for our analysis pipeline is as follows, unless stated otherwise stated:

**Pruning** Pruning the taxa associated with a random subtree, as well as removing the sequences from the MSA,

**Alignment** Realigning the pruned sequences with respect to the remaining reference MSA,

**Damage** Fragmenting and deaminating the aligned sequences,

**Placement** Running various placement programs on the pruned sequences,

**Assessment** and Computing and summarizing the placement error.

**Fig. 1.**
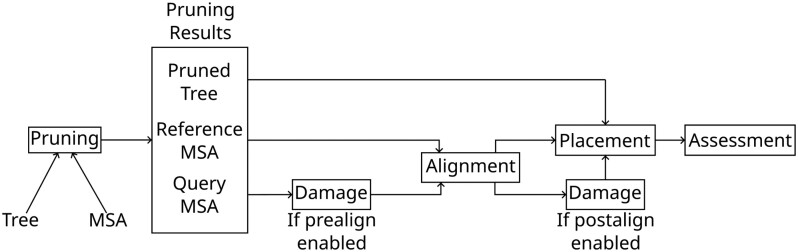
Diagram of the modified PEWO pipeline. The steps “Pruning,” “Alignment,” “Damage,” “Placement,” and “Assessment” are described in Section “Simulating Ancient DNA Damage”.

We present a graphical outline of these steps in Fig. 1. The full pipeline is available on GitHub at https://github.com/computations/PEWO.

### Simulating aDNA Damage

To simulate the characteristic *postmortem* damage that DNA undergoes as it ages, we implemented the Briggs Model of DNA damage (Briggs et al. 2007) in a tool we call PyGargammel. The primary use case of PyGargammel is to take a FASTA file with sequences and insert aDNA typical damage. This model has 4 parameters that control the effects and magnitude of aDNA-like sequence damage. Using the same notation as Briggs et al. (2007), the parameters are:

- The probability of nicks 0≤ν≤1,
- the parameter for the geometric distribution from which the overhang length is drawn 0<λ≤1,
- the probability of deamination in single-stranded regions $0\leq \delta_{ss}\leq 1$,
- and the probability of deamination in double-stranded regions $0\leq \delta_{ds}\leq 1$.

The first mechanism in the model determines the probability of breaking (nicking) ν, for each base. These reads then have an overhang with a length that is drawn from geometric distribution with success probability λ. Which side of the reads (5′ or 3′) the overhang is located on depends on numerous factors that occur during sequencing. However, for the purpose of damage simulation, it is essential that the overhang is present only on the 5′ or the 3′ side of the read. Therefore, when simulating the overhang, we randomly generate an overhang on the 5′ side for 50% of reads, and generate an overhang on the 3′ side for the remaining 50% of the reads. Then, deamination damage is applied with a probability that depends on whether the base is in a single or double-stranded region. If the base is in the overhang region, then the single-stranded deamination rate $\delta_{ss}$ is used to determine the deamination probability. If the base is not located in an overhang region, then the double-stranded deamination rate $\delta_{ds}$ applies.

Note that there are 2 distinct types of possible deamination damage, C→T and G→A when using double-stranded libraries. C→T damage is the direct result of a cytosine being deaminated (this is to say, losing a part of its chemical structure), which results in the base being misincorporated as a T. However, G→A damage is *not* a direct result of deamination. Instead, it is a consequence of the genomic library preparation for the sequencing process. More specifically, when the sample is prepared for sequencing, overhangs are repaired, which transforms the C→T damage into G→A damage on the complementary strand. In this case, the overhang is repaired with an A instead of a G, as G is the complementary base to a deaminated C. If the sequence is read from left to right, then *only*  C→T damage can occur in the read if the overhang is on the left side, and *only*  G→A damage occurs if the overhang is on the right side.

PyGargammel relies on this model and implements it as follows: It starts by reading a FASTA file and iterates over each sequence in the file by performing the following steps:

1. Iterate through bases and mark nicks with probability ν.
2. Break the sequence into reads at the nick points.
3. Compute overhang lengths on both ends with distribution Geom(p=λ).
4. Perform deaminations (C→T and G→A damage)
  - If the read has an overhang (with a possible length of zero) on the 5′ end, and the base is in the overhang region, apply C→T damage with probability $\delta_{ss}$. If the base is not in the overhang region, apply C→T damage with probability $\delta_{ds}$.
  - Likewise, if the read has an overhang (with a possible length of zero) on the 3′ end, and the base is in the overhang region, apply G→A damage with probability $\delta_{ss}$. If the base is not in the overhang region, apply damage with probability $\delta_{ds}$.
5. (Optional) Position the read such that it is aligned with its original position in the original sequence.
6. (Optional) Filter sequences that are too short if a minimum read length was supplied.
7. (Optional) If a minimum read count threshold was specified, repeat the process from Step 1 accumulating additional reads until the threshold has been achieved.
8. (Optional) If a maximum number of reads was specified and too many reads were generated, downsample uniformly at random to the provided threshold.

It is important to discuss how PyGargammel handles ambiguous characters and missing data, as empirical MSAs often have ambiguous characters, which can affect the results of downstream analysis. As PyGargammel is designed to also operate on already aligned data, there might be gaps present in the sequences to be damaged. The resulting reads need to be still in alignment after processing, so gaps and ambiguous characters are retained as the respective sequence is being processed. This means that gap characters will appear in the resulting damaged reads, and gaps will also affect the damage distribution, as gap characters cannot undergo either C→T or G→A damage. However, PyGargammel will completely remove reads entirely consisting of either fully ambiguous or gap characters. In addition, when a minimum read length is specified, only nongap sites are counted to determine read length.

Finally, if a reference alignment is provided, PyGargammel will reject reads with no nongap characters in common, a technique known as premasking. We believe this choice is justified, as such reads would be difficult to align and therefore difficult to classify by any method.

We verified that PyGargammel is producing output that is in accordance the model, and this verification is presented in the Supplementary material online.

### Pipeline Parameters

In order to fully investigate the impact of aDNA damage on phylogenetic placement accuracy, we perform a grid search over appropriate damage simulation parameter intervals based on the estimated values from Briggs et al. (2007) for each parameter. The complete grid was evaluated to investigate the independent effect of each damage simulation parameter on phylogenetic placement accuracy. The specific values (grid points) we tested are provided in the Supplementary material online. Note that, if we equally increase the number of investigated values per parameter, the number of grid points we need to evaluate grows according to $O(n^{4})$. Hence, the number of grid points we need to evaluate increases too rapidly in order to be computed in reasonable time. Efficiency can be improved upon by investigating the behavior and impact of a single parameter at a time while keeping the remaining parameters fixed.

An initial parameter space exploration on a single dataset showed that the nick frequency is the most important parameter (w.r.t. its impact on placement accuracy). Hence, we only thoroughly investigate the effects of the nick frequency parameter. To this end, we sample ν values ranging from 0.0 to 0.03. The value of 0.03 slightly exceeds the value estimated in Briggs et al. (2007), which was found to be ν=0.024 for Neanderthal samples. Overall, we assessed 9 ν values, with a higher value density near 0.0, as this is the parameter region where placement accuracy is most sensitive to slight changes in ν. In Table 4, we show both, the theoretical, and the actual median read length for each value of ν in order to better illustrate the impact of changing ν on read length. Noticeably, the largest reductions in both, estimated, and actual median read length stem from values near 0.0. The fragment lengths for the estimated values and the actual values differ due to the original sequence being of finite length, as the maximum length of any read generated by PyGargammel is limited by the original sequence length. Furthermore, we also exclude reads with < 15 bp (see further below for a rationale), which further skews the actual values.

Beyond ν, we experimentally determined that the overhang parameter λ is the second most important parameter, again with respect to its impact on placement accuracy. As before, we base the grid value intervals on the values listed in Briggs et al. (2007). Since overhangs are modeled as iteratively “growing” with a probability λ of the process terminating at the current overhang length, the parameter value associated with no damage is 1.0. This occurs when the process terminates immediately, resulting in a zero length overhang. Correspondingly, the overhang lengths increase as the parameter value decreases. We tested 6 values with the most extreme damage being 0.15, which is slightly higher than the value estimated in Briggs et al. (2007).

The deamination rates $\delta_{ss}$ and $\delta_{ds}$ were both found to induce a minor damage effect during initial experiments. Therefore, we only consider 2 values for $\delta_{ss}$ and 3 values for $\delta_{ds}$ in our grid. For $\delta_{ss}$, the upper interval bound follows, again, Briggs et al. (2007). On the other hand, the upper $\delta_{ds}$ value is a very extreme value of 0.1, which is far greater than the value found in Briggs et al. (2007). We included this extreme value because we intend to investigate the effects of high levels of C→T and G→A damage on placement accuracy, as this is the unique feature that yields aDNA damage being different from “just” very short reads. In particular, we explore placement accuracy for damage rates that are substantially higher than expected in order to investigate the worst-case effect of misincorporation on placement accuracy.

Finally, we implemented 2 additional constraints on the simulated reads. The first is that a minimum of 10 reads per selected sequence from the reference MSA is generated. This ensures that we have a sufficient number of reads to compute proper summary statistics. The second is a minimum read length of 15 bp. This mimics representative analyses of aDNA samples, where reads below a certain length (typically 30 bp) are excluded in a prefiltering step.

### Analysis

We ran PEWO with the following placement tools: RAPPAS (Linard et al. 2019), EPA-Ng (Barbera et al. 2019), pplacer (Matsen et al. 2010), and APPLES (Balaban et al. 2020). We do not explore different placement program options (e.g. various heuristics) because initial experiments did not exhibit substantial placement accuracy differences as a function of these options. Therefore, in order to also economize on computational resources, we elected to use one set of placement program options for all runs. These options are described in Table 2.

**Table 2:** Tool option and heuristics used for all datasets and parameter values

Tool	Options and Heuristics
RAPPAS	k = 7, omega = 2.0, reduction = 0.99
EPA-Ng	h1, g = 0.99999
pplacer	max-strikes = 6, strike-box = 3, max-pitches = 40
APPLES	OLS, MLSE

Experiments were performed on a server with 40×86 CPU cores and 754 GiB of memory. The number of independent subtree prunings and accuracy evaluations (denoted as a “pruning” in PEWO) was set to 10 for all datasets (specifically a dual socket server with 2 Intel Xeon Gold 6,148). This is to say, that 10 separate experiments were conducted for each set of parameters on each reference MSA and the results were aggregated. PEWO was run with the Snakemake option -cores 40.

To better visualize our experimental results, we fit a linear model augmented with cubic splines for each parameter against the eND. The eND is computed as LWR(p)×d(p), where LWR(p) is the likelihood weight ratio of the placement, and d(p) is the node distance between the estimated placement and the true placement. We use eND as it better accounts for uncertainty in the specific placement location. We added splines to the model in order to capture the decreasing marginal impact of the damage model parameters. The regression was performed using R, with the command geom_smooth(method=“lm”, formula = y ~ splines::ns(x, *k*)), where *k* is the number of splines used for the regression on the specific parameter. We used k:=3 for the regression on ν and λ, k:=2 for the regression on $\delta_{ds}$, and k:=1 for the regression on $\delta_{ss}$. We vary the number of splines because the number of parameter values sampled varies between parameters.

Finally, the summary statistics about the prunings which PEWO generated for each dataset is presented in the Supplementary material online.

### Additional Tests

In addition to the results presented here in the main text, we also investigated 2 additional factors relating to placement performance when using aDNA data. First, we investigate the effects of GC content on placement accuracy and if GC content affects the relative importance of aDNA damage parameters. Second, we investigate the choice of alignment tool used to align the query sequence to the reference sequence. As mentioned above, the results presented here in the main text use HMMER to align query sequences to the reference alignment. Although we also investigated the performance of other alignment tools, we found that HMMER yielded the best performance, and so we have restricted experiments in the main paper to use only HMMER.

The results of both of these additional tests are presented in the Supplementary material online.

## Results

We display the regressions describing placement accuracy as a function of different simulation parameter values in Fig. 3. Additionally, we show the scaled parameter coefficients for a linear model between in Table 3. We found that the primary factor affecting placement accuracy is the nick frequency parameter ν. For example, when pooling experiments from all datasets together, the regression yields ν=0.2321 vs the next largest coefficient $\delta_{ds}=0.015$. Additionally, ν is the only parameter, which is statistically significant at the p=0.01 level for all datasets. The parameter $\delta_{ds}$ had the second most substantial impact on placement accuracy and is also statistically significant for all datasets except ds04. However, it is still at least an order of magnitude smaller when compared with the effect of ν.

**Table 3:** Table of scaled coefficient values for the the linear model $\text{eND}∼\nu +\lambda +\delta_{ds}+\delta_{ss}$ for each dataset, as well as the regression for all datasets

	(Scaled) Coefficients			
Dataset	ν	λ	$\delta_{ds}$	$\delta_{ss}$
ds01	$2.546\times 10^{-1}$	$-1.016\times 10^{-2}$	$4.538\times 10^{-2}$	$4.75\times 10^{-3}$
ds02	$3.649\times 10^{-1}$	$2.256\times 10^{-3}$	$1.83\times 10^{-2}$	$-3.033\times 10^{-3}$
ds03	$3.77\times 10^{-1}$	$2.617\times 10^{-3}$	$3.682\times 10^{-2}$	$5.453\times 10^{-3}$
ds04	$4.099\times 10^{-1}$	$2.513\times 10^{-3}$	$2.692\times 10^{-3}$	$-4.519\times 10^{-3}$
ds05	$3.568\times 10^{-1}$	$-1.194\times 10^{-3}$	$1.616\times 10^{-2}$	$1.837\times 10^{-3}$
ds06	$4.282\times 10^{-1}$	$1.903\times 10^{-3}$	$1.017\times 10^{-2}$	$-5.61\times 10^{-3}$
ds07	$3.058\times 10^{-1}$	$-6.758\times 10^{-3}$	$6.97\times 10^{-2}$	$1.064\times 10^{-2}$
All	$2.321\times 10^{-1}$	$-1.319\times 10^{-3}$	$1.538\times 10^{-2}$	−4.733×10−4

Bold values are significant at the p=0.01 level.

**Table 4:** Table of estimated and actual read lengths based on the Briggs model

	Median read length	
ν	Estimated	Actual
0.0000	∞	3,409
0.0001	6,931	3,388
0.0005	1,385	1,309
0.001	692	736
0.005	138	165
0.01	68	91
0.02	34	53
0.025	27	45
0.03	22	40

The median is computed for each dataset. The values differ between the estimated values and the actual values due to the original sequence being finite in length, as the maximum length of any read generated by PyGargammel is limited by the original sequence length.

The remaining parameters had a negligible effect on placement accuracy. The parameters λ and δ_*ss*_ are generally not statistically significantly different from 0.0, with the only exceptions being λ for ds01 and ds02. To further investigate this, we computed the error rate for generated reads. In Fig. 4, we show the error rate as a histogram for datasets with $\nu =0.025,\lambda =0.15,\delta_{ss}=0.65$ and $\delta_{ds}=0.015$. Most reads exhibit a relatively small number of errors (the percent number of sites in a read that differ from the source sequence), with a median error rate of 4%.

**Fig. 2.**
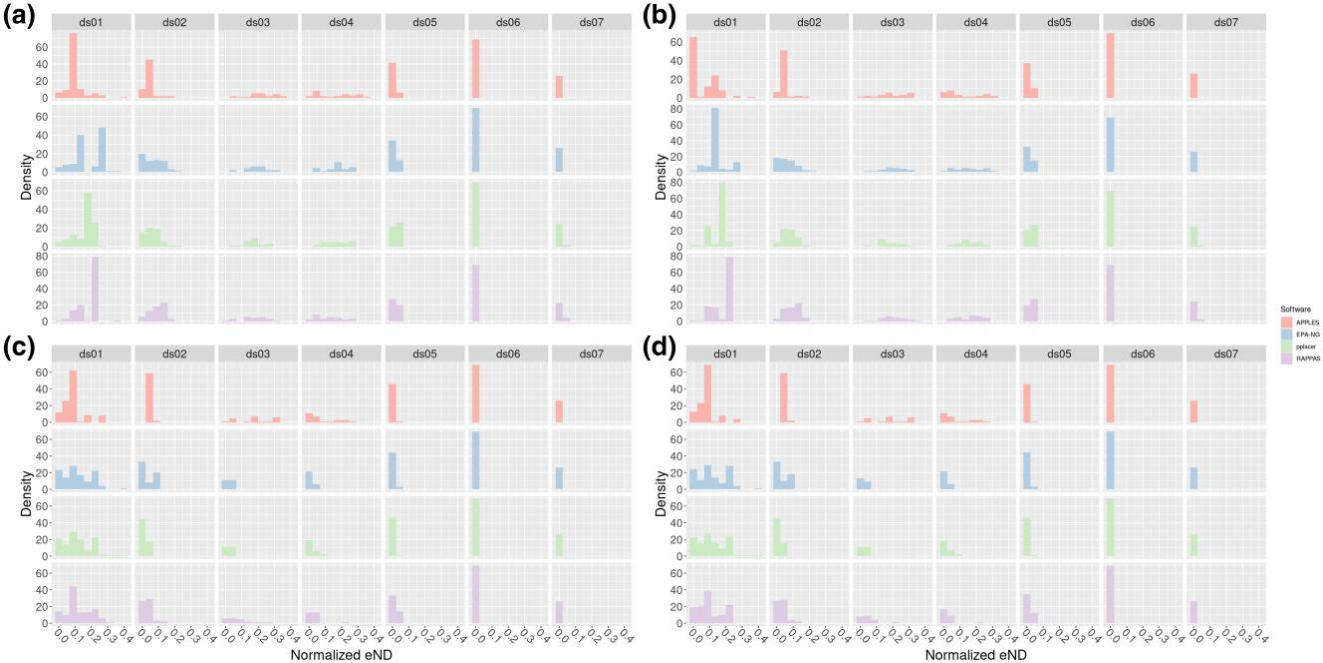
Histogram of normalized effective node distances for all datasets. In addition, each subfigure has been faceted by the respective placement software being used. Top plots (a and b) have nicks enabled (ν=0.025), and bottom plots (c and d) have nicks disabled (ν=0.0). Plots on the left (a and c) have other forms of damage enabled ($\lambda =0.15,\delta_{ss}=0.65,\delta_{ds}=0.015$). Plots on the right (b and d) have other forms of damage disabled ($\lambda =1.0,\delta_{ss}=\delta_{ds}=0.0$). All plots share the same scale for the *x* axis. Parameters for enabled damage were chosen due to their similarity to the inferred values in Briggs et al. (2007).

To better illustrate the effect of a high nick rate on placement accuracy, we plotted an eND histogram that is normalized by tip count in Fig. 2. In both plots, subplot **a** shows the distribution of normalized eND for all damage *enabled* (that is, parameter values of $\nu =0.025,\lambda =0.15,\delta_{ss}=0.65,$ and $\delta_{ds}=0.015$). Subplot **b** shows the distribution of normalized eND with nick damage *enabled*, but other forms of damage *disabled* (parameter values $\nu =0.025,\delta_{ss}=\delta_{ds}=0.0,$ and λ=1.0). Subplot **c** shows the distribution of normalized eND for nick damage *disabled*, but other forms of damage *enabled* (parameter values same as subplot a, but ν=0.0). Subplot **d** shows the distribution of normalized eND for all forms of damage *disabled* (parameter values $\nu =\delta_{ss}=\delta_{ds}=0.0$ and λ=1.0). We also present the medians for each of these histograms in the Supplementary material online. To properly summarize the results, we report some selected summary statistics. For ds04, the median normalized eND for all software in subplot **a** is 0.16; in subplot **b** the median normalized eND is 0.15; and in subplots **c** and **d** the median normalized eND is 0.0. For emphasis, we reiterate that subplots **a** and **b** have nick damage *enabled*, whereas subplots **c** and **d** have nick damage disabled.

**Fig. 3.**
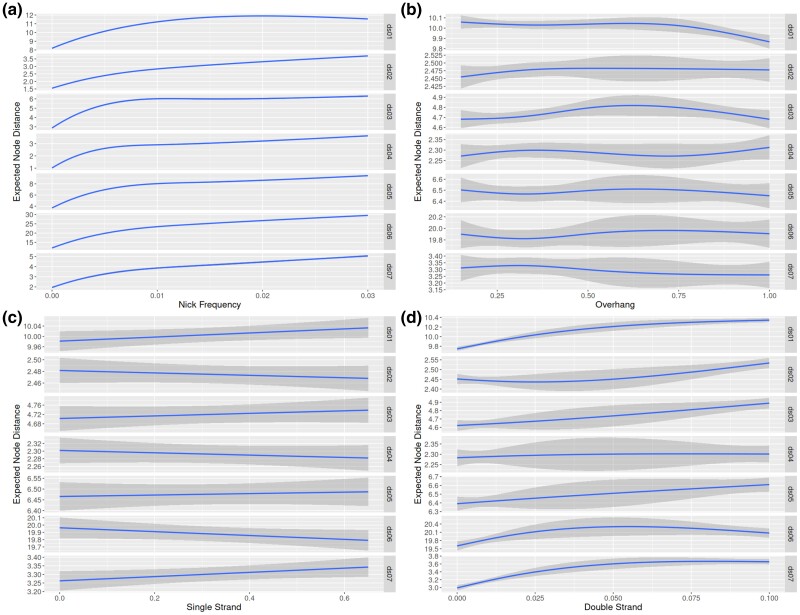
Plot of regressions against parameters for each dataset. Please note that the y scale varies for each dataset. Each regression is against the eND. The subfigures **a**, **b**, **c**, and **d** plot ν, λ, $\delta_{ss}$m, and $\delta_{ds}$ against eND, respectively. Plot generated using ggplot2 with geom_smooth(method=“lm”, sd=TRUE, formula = y ~ splines::ns(x, *k*)), where *k* varies with the dataset. k=3 for plots **a** and **b**, k=2 for plot **d**, and k=1 for plot **c**.

**Fig. 4.**
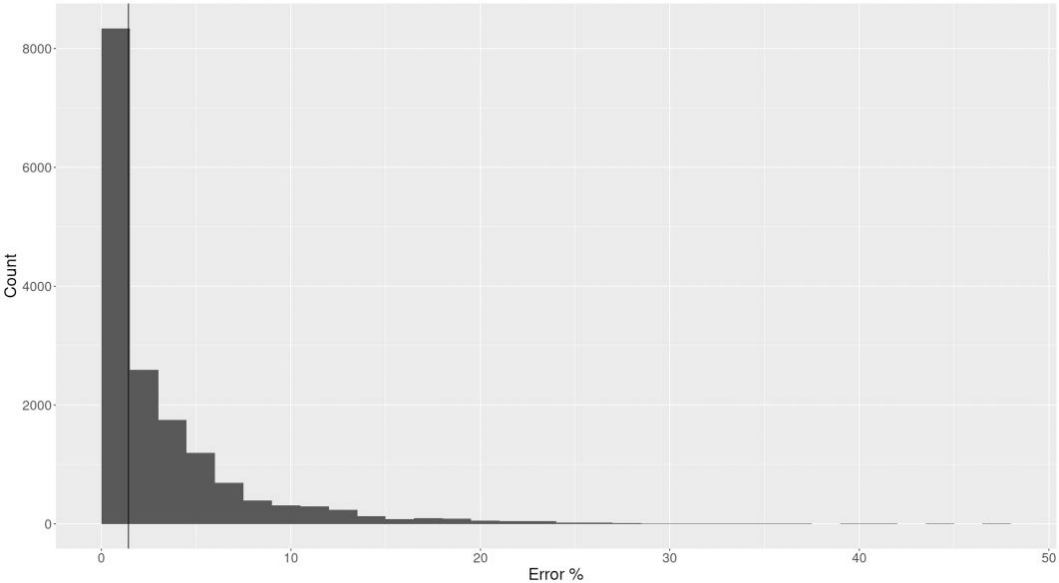
Histogram of percent of error sites per read for reads generated under the model parameters ν=0.025, λ=0.15, $\delta_{ss}=0.65,$ and $\delta_{ds}=0.015$. An error site is a site which has either experienced C→T or G→A damage. A vertical line is drawn at the median, where 1.4% of sites are errors. The max percent error for all reads is 47%.

In Fig. 2, we can see a clear shift to the left between the top and bottom (that is, the subplots **a** and **b** vs **c** and **d**). This shift to the left indicates an average improvement in placement accuracy. In contrast, we do not observe a similar shift between the left (other damage types enabled) and right (other damage types disabled) plots. The median normalized eND for each tool and dataset, shown in the Supplementary material online, also show this. For example, the median normalized eND for EPA-Ng is larger in either subplots **a** or b (nicks enabled) when compared with subplots **c** or **d** (nicks disabled) for all datasets.

Over all datasets, EPA-Ng performed the best, both in high damage scenarios and in low damage scenarios. For example, EPA-Ng performed the best at placing sequences damaged using the parameters in subplot **a** for 3 out of 7 datasets, whereas the next best tool APPLES performed the best in 2 out of 7 datasets. The medians for each histogram are shown in supplementary table 6, Supplementary Material online. Most tools experienced degraded performance in a high damage scenario (subplot **a**) when compared with no damage scenarios (subplot **d**). For example, in ds04 in a high damage scenario EPA-Ng placed query reads with median normalized eND of 0.17, but in a no damage scenario EPA-Ng placed query reads with a median normalized eND of 0.0. Similar results are found for most tools in all datasets. The exception to this performance loss is APPLES, which generally suffered only small performance losses in high damage scenarios. In ds02, for example, APPLES performed nearly the same in a high damage vs no damage scenario (0.05 median normalized eND in both cases).

Similarly, RAPPAS performed better or at least as well in subplot **b** when compared with **a** for nearly all datasets, with ds06 being the exception. That is, RAPPAS performs better *with* nicks enabled than without in the presence of other forms of damage. For example, this is particularly pronounced in ds04, where the median normalized eND in subplot **a** is 0.1148, and in subplot **b** it is 0.1729. However, for other datasets, the difference between nick enabled and nicks disabled is negligible. For example, RAPPAS achieved a median normalized eND of 0.09139 in subplot **a**, and 0.09579 in subplot **b**.

## Discussion

Based on our analysis, the primary factor influencing aDNA placement accuracy is the nick frequency, or equivalently the read length. Furthermore, the effects of deamination errors are minor, confirming our repeated empirical observations that likelihood-based models as implemented in EPA-Ng are generally robust with respect to noise and sequencing error.

One way to ameliorate the placement error from aDNA damage is to incorporate the aging process as an additional model process. Currently, none of the placement tools tested here do this. However, the largest contributor to placement error when analyzing aDNA data is the amount of information available in the sequence to be placed. More simply, read length is the primary driver of error for aDNA reads. When reads become shorter, the probability of correctly placing the reads decreases (e.g. Fig. 3). The reason for this becomes clear if we examine the distribution of read lengths as shown in Table 4. Both the estimated and actual median read lengths rapidly decrease as ν increases. This reflects the increase in placement error shown in Fig. 3**a** with increasing nick frequency.

In contrast, the total amount of deamination events (that is, C→T and G→A damage) is not as important for placement accuracy. As we have shown in Fig. 4, the rate of site errors is relatively low compared with the length of the read, even for higher damage rates. This is expected, as only 2 bases (C and G) are available to being misincorporated. On a typical “random” sequence, only 50% of sites will be eligible to be damaged. Furthermore, in practice GC content is often below 50%. For our empirical datasets, GC content varies between 17% and 43%. Of course, the so-called GC-content varies among species and genomic regions. Hence, we would expect that sequences will be more difficult to place correctly as a function of their GC-content. However, we investigated the effect of increasing amounts of GC-content on placement accuracy in the presence of aDNA damage using simulated data, and found no major differences between high and low GC-content datasets. Details of this analysis are described in the Supplementary material online.

The reasons for the unexpected performance of RAPPAS between the damage scenarios depicted in Fig. 2 subplots **a** and **b** are unclear. Specifically, we would expect all tools to perform worse in the presence of nicks, but RAPPAS often performs better when all damage is enabled, compared with when all damage but nicks are enabled. However, on datasets ds02, ds03, ds05, and ds07 RAPPAS is more accurate in the nicks enabled case (Fig. 2 subplot b.) We assume that since RAPPAS is the only *k*-mer based tool we tested, we are likely observing a behavior that is specific to *k*-mer based approaches. Potentially *k*-mers are more sensitive to point errors with increasing read length, as the uncertainty of short reads might allow them to better tolerate point errors. This might be due to the fact that a shorter read has fewer (in number) *k*-mers, which translates to lower overall placement confidence, when compared with a longer read. Therefore, when placing long damaged reads with a *k*-mer method, the number of *k*-mers may indicate a high confidence, but the error leads the actual placement astray.

Some datasets are more challenging to perform accurate placement in the presence of damage than others. For example, all tools performed well (median normalize eND under 0.01) with damage enabled for datasets ds06 and ds07, whereas all tools performed poorly (median normalized eND over 0.1) for datasets ds01, ds03, and ds04 under the same damage scenario. This difference can be partially attributed to being an artifact of the normalization procedure, since having a small number of taxa increases the impact of small errors. However, that cannot explain the difference in performance between ds01, where all tools performed poorly (above 0.1 median normalized eND) and ds02, where all tools performed moderately well (below 0.1 median normalized eND.) It is likely that ds01 constitutes a challenging dataset to place accurately, even under a scenario without damage. This is evidenced by nearly all tools performing poorly on ds01 without damage (median normalized eND exceeding 0.1 for tools other than APPLES. APPLES achieved a median normalized eND of 0.0702.) Additionally, all tools performed well, when compared with ds01, on ds02 (median normalized eND < 0.05.) Together, this indicates that the difficulty of the underlying placement problem has a strong influence on placement performance in the presence of damage. This is to say, the difficulty of placement in the presence of aDNA damage is proportional to the difficulty of placement without damage.

While we based our investigation around parameters found in Briggs et al. (2007), which is based on reads obtained from Neanderthal samples, the parameter values tested also cover other data sources, such as mollusks (Pedersen et al. 2015). Additionally, further investigation into some of the parameters is unlikely to clarify the issue any further, especially for the nick frequency ν. At the maximum value of ν in this study, many of the reads generated were filtered by the already permissive (w.r.t. to current practice in aDNA studies) minimum read length cutoff at 15 sites. Further increases in ν would only marginally decrease the realized read lengths, as most reads would be filtered by the minimum read length.

We find that our primary results, that nick frequency is the dominant cause of placement error, still hold when including errors induced by aligning the reads using a selection of representative and widely used alignments tools. In supplementary table 6, Supplementary Material online, we show regression coefficient values for ν, λ, δ*_ds_*, and δ*_ss_*. We find that when using various alignment tools, nick frequency is the most impactful parameter by one order of magnitude. This is visualized in supplementary figure 3, Supplementary Material online. Therefore, the choice of alignment tool does have an impact on the final accuracy of placement results, particularly under high damage scenarios. So-called alignment extension methods, which treat the reference alignment as fixed, and only align the reads relative to the reference alignment, are the most accurate in terms of alignment error. HMMER, using an alignment extension method, performed the best among all alignment tools we tested (median eND of 1.81 vs 2.04 for the next best tool MAFFT.) We assume that the reason for this is that inferring the reference sequence alignment that comprises substantially more sequence data, is generally substantially easier than aligning short aDNA reads and longer high quality reads simultaneously. Therefore, treating the reference alignment as fixed is likely to yield, as shown by our experiments, more accurate results compared with standard de novo MSA consisting of the reference sequences and the aDNA reads. However, there are limitations to this analysis, which we discuss later.

All of this together indicates that phylogenetic placement is an appropriate tool for analyzing aDNA sequences. While the frequency of nicks strongly affects the probability of accurately placing reads, this is predominantly due to length of the reads to be placed, and not due to other damage types. Importantly, *all* methods to identify aDNA reads will experience difficulties with extremely short reads, as the amount of information contained within a read is directly proportional to its length. Furthermore, placement seems to be robust to other forms of aDNA damage, even when reads are short (see Fig. 2).

There are some limitations to this work. First, we did not examine the case where C→T damage occurs in both overhangs instead of G→A in the second overhang, as is the case in single-stranded libraries. However, we do not expect this to have any effect on the results presented here, as it is likely the number of misincorporations that matters, and not the type. Additionally, while we did investigate different alignment tools in the Supplementary material online, we did not thoroughly examine the effects of alignment on placement accuracy. Notably, we did not directly assess read alignment accuracy but only considered placement accuracy of the aligned reads, as a form of downstream accuracy. A thorough exploration of alignment tools with respect to aDNA damage should score alignment tools based on alignment accuracy as opposed to a downstream proxy value, as the final placement error might be induced by either the alignment tool, the placement tool, or some challenging-to-assess interaction between them. Hence, it is currently unclear if aDNA damage-aware alignment methods can yield higher accuracy than standard alignment methods.

Finally, our work indicates that a dedicated aDNA damage-aware phylogenetic placement model is unlikely to substantially improve results.

## Supplementary Material

msaf006_Supplementary_Data

## Data Availability

The modified PEWO pipeline is available at github.com/computations/PEWO. Datasets, result files, plotting scripts, and utility scripts used can be found on Dryad at https://doi.org/10.5061/dryad.4f4qrfjn3.
