# Supplementary material for: Read Length Dominates Phylogenetic Placement Accuracy of Ancient DNA Reads
Source: Mol Biol Evol. 2025 Jan 17;42(2):msaf006. doi: 10.1093/molbev/msaf006 (PMC11839404; doi:10.1093/molbev/msaf006)
Supplement: msaf006_Supplementary_Data [file msaf006_supplementary_data.pdf]

Supplemental Material for "Read Length Dominates Phylogenetic Placement Accuracy of Ancient DNA Reads"

Ben Bettisworth Nikolaos Psonis Nikos Poulakakis Pavlos Pavlidis
Alexandros Stamatakis

January 13, 2025

1 Modeling Expected Node Distance as a Function of aDNA damage parameters

In order to investigate which parameter in the Briggs' Model has the largest impact, it is natural to fit a linear model, and examine the relative magnitude of the (scaled) coefficients. Explicitly, this model is

$$\text{eND} \sim \nu + \lambda + \delta_{ds} + \delta_{ss}. \tag{1}$$

However, in the Briggs' Model, λ only controls the length of the overhang, which is not directly observed. Instead of directly influencing the amount of damage that occurs in a read, λ controls the relative impact of the two deamination parameters: δ_{ds} and δ_{ss}. When λ is equal to 1.0, the overhangs are short, and deamination occurs at the rate δ_{ds} for all sites. As λ decreases to 0.0, the length of the overhangs increase, and more sites are deaminated at rate δ_{ss}. It is clear then that λ controls the relative impact of δ_{ds} and δ_{ss}. Therefore, it is reasonable that instead of simply modeling λ as an additive term we model the contribution of λ as modifying δ_{ds} and δ_{ss}. To accomplish this, we implement an alternative model as

$$\text{eND} \sim \nu + \lambda \times \delta_{ds} + (1 - \lambda) \times \delta_{ss}. \tag{2}$$

In our analysis, we will use both of the models in Eq. 1 and 2. We will present the results of a regression for both models here in the supplementary material for completeness, but in the main text we will only present the results for the model presented in Eq. 1, as the goodness-of-fit for both models is very similar (0.05408 vs 0.05399).

2 Investigating the effects of GC content on placement error in the presence of aDNA damage

In order to be sure that our results are not driven by our utilization of GC-poor datasets, we simulated datasets with varying levels of GC content. To generate the sequences we generated random trees with 300 tips via a custom tool which uses a random coalescent process (similar to `rcoal`. Using this random tree, we used AliSim (Ly-Trong et al., 2022) to generate a simulated sequence using the following command.

```
iqtree2
    --alisim <PREFIX>
    -t <RANDOM TREE>
    --out-format fasta
    --length 5000
    -m GTR+G+I+F
      {(.5 - .05*i)/(.05 * i)/(.05 * i)/(.5 - .05 * i)}
```

This simulates a 5000 b.p. sequence under the GTR model (with equal rates), 4 gamma rate categories and invariant sites. Initial frequencies are based on the dataset name, where `i` is the value of `sds0i`. For example, `sds03` has initial frequncies of `{(0.5 - 0.05 * 3) / (0.05 * 3) / (0.05 * 3) / (0.5 - 0.05 * 3)}` which is equal to `{0.35 / 0.15 / 0.15 / 0.35}`. Each data dataset was run using the modified PEWO pipeline as described in Section 2 of the main text, except that the tool `pplacer` was skipped due to technical difficulties. Results from these runs are summarized in Suppl. Figure 1. The regressions parameters are the same as in Figure 4 of the main text.

As it can be seen Suppl. Figure 1, the GC content of the data does not impact the results.

Dataset	Initial Frequencies	Realized GC%
sds01	`{.45 / .05 / .05 / .45 }`	10.1%
sds02	`{.40 / .10 / .10 / .40 }`	19.9%
sds03	`{.35 / .15 / .15 / .35 }`	29.9%
sds04	`{.30 / .20 / .20 / .30 }`	39.9%
sds05	`{.25 / .25 / .25 / .25 }`	50.0%
sds06	`{.20 / .30 / .30 / .20 }`	60.0%
sds07	`{.15 / .35 / .35 / .15 }`	69.9%
sds08	`{.10 / .20 / .20 / .10 }`	80.0%
sds09	`{.05 / .45 / .45 / .05 }`	90.0%

Supplementary Table 1: Datasets, the initial frequency parameters used for simulation, and the realized GC% of each dataset.

The major conclusion of the main paper, that read length is the single most important factor impacting phylogenetic placement accuracy, is unaffected by the GC content of the data.

3 Determining Tool Parameters

Prior to evaluating all datasets for all parameters, we performed an initial exploration using ds06. As part of this initial investigation, we investigated whether or not the tool parameters have an substantial effect on placement accuracy with regard to ancient DNA damage. To this end, we ran each tool with a variety of parameters (specified in Suppl. Table 2) on ds06 with 10 prunings. We also varied the aDNA damage parameters, using the same parameter set as in the main paper. For each tool parameter set, computed a regression with Eq. 1 and recorded the estimated coefficient. These coefficients are summarized in Suppl. Figure 2.

The results of this exploration show that, for the purposes obtaining regression coefficients (alternatively, assessing the relative importance aDNA damage factors), tool parameter choice is largely unimportant. For APPLES, EPA-NG, and `pplacer` the choice tool parameter produces the same conclusion: that ν is the most impactful parameter on placement accuracy when analyzing aDNA damage. The exception to this is RAPPAS, which had large variation in the estimated coefficient for ν. This indicates that tool parameter choice does matter, and if we wish to limit our RAPPAS evaluations to one parameter set, we must choose our parameter carefully.

To this end, we examined the relative performance of RAPPAS with different tool parameters in terms of median eND, and found that the parameter set $k = 8, \omega = 2.0$, and `red`$= 0.99$ gave the

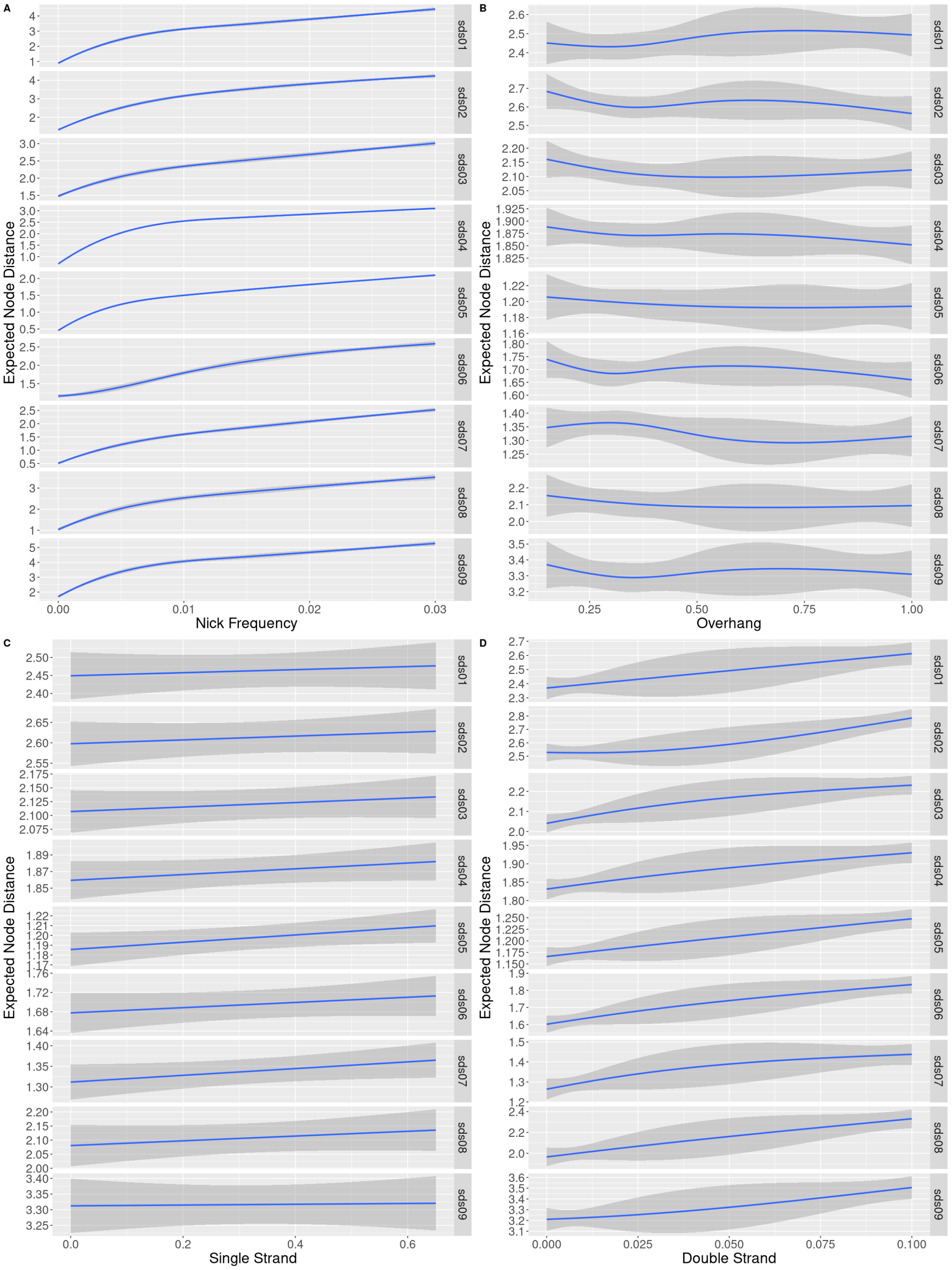

Supplementary Figure 1: Regressions for model parameters using simulated data with varying levels of GC content. GC content is minimized in sds01 (GC% of 10%), and increases by steps of 10% until it is maximized in sds09 (GC% of 90%). Sequences were simulated using AliSim (Ly-Trong et al., 2022).

Tool	Parameter	Values Tested
APPLES	Method	`OLS`, `FM`, `BE`
EPA-NG	Heuristic	`h1`, `h2`, `h3`, `h4`
	g	0.5, 0.8, 0.9999
	G	0.1, 0.2, 0.5
`pplacer`	Max Strikes	6, 7, 8
	Strike Box	3, 4, 5
	Max Pitches	40, 50, 60
RAPPAS	k	6, 7, 8
	ω	1.5, 2.0
	`red`	0.5, 0.8, 0.99

Supplementary Table 2: Parameters used determine the effect of tool parameters on placement accuracy in the presence of aDNA damage. Note that for EPA-NG, g is only used with `h1`, and G is only used with `h2`.

k	ω	`red`	Median eND
7	2.0	0.99	34.1
8	2.0	0.99	31.4

Supplementary Table 3: Table showing the best performing parameter set for RAPPAS and the parameter set used in the main paper.

best placement results. However, k controls the size of the k-mers that RAPPAS uses to perform placement, and the computational cost with $k = 8$ compared to $k = 7$ is much substantially more expensive. Therefore, we elected to instead test RAPPAS with $k = 7$, with other tool parameters kept equal, in order to save on compute time, as RAPPAS evaluations with $k = 7$ gave similar results to $k = 8$, as can be seen in Suppl. Table 3. We further justify this choice by noting that we are not looking to evaluate the relative performance of placement tools, but instead to investigate the effects of aDNA damage on placement accuracy.

4 Evaluating alignment tools

In order to investigate the performance of different alignment tools with respect to placement accuracy, we implemented a separate Snakemake pipeline, which can be found at `https://github.com/computations/alignment-pipeline`. This new pipeline is similar to the pipeline described in the main paper, with the major difference being that we align the sequences with

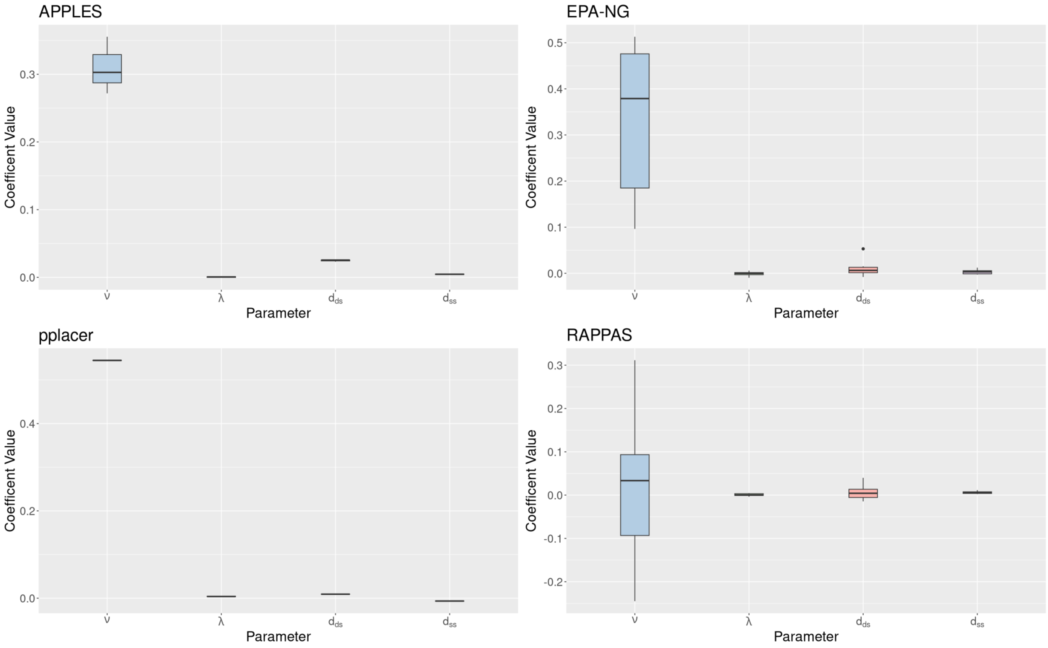

Supplementary Figure 2: Boxplot of the regression value for each of the aDNA damage parameters using the model in Eq. 1 for various tool parameters.

several different tools, and we apply the simulated damage to the query sequences *before* alignment. To compute the alignment, we chose 4 widely used alignment tools: HMMER Eddy (2011); Muscle5 Edgar (2022); MAFFT Katoh et al. (2002); and Clustal Omega (hereafter ClustsalO) Sievers et al. (2011).

In addition to the aforementioned alignment tools, we also placed sequences which had perfect alignment with the source MSA. Normally, the sequences from PyGargammel are produced as unaligned reads. However, using information which was recorded during the damage simulation, we can precisely realign the reads to the source location by inserting the corresponding number of gap characters before and after the read. An important difference between the "null" alignment, and the alignment method used in the main pipeline is that sequences are modified by an alignment tool. We included this so-called "null" alignment such that we can separate the error from phylogenetic placement itself, and placement error induced by errors from the alignment process. As a final point, the "null" alignment should provide a floor for placement accuracy, and all error in the "null" alignment is due errors in the placement inference.

The pipeline implemented is:

1. Simulate a tree using IQ-TREE with 100 taxa;
2. Simulate an alignment with 1000 sites using the simulated tree under the GTR model with Alisim Ly-Trong et al. (2022);
3. Prune a subtree with at least 3 leaves;
4. Split simulated alignment into reference and query sequence subsets based on the pruned subtree;
5. Randomly generate random damage parameters (see text for details);
6. Damage query sequences according to generated parameters;
7. Align reference sequences with damaged query sequences with MAFFT, ClustalO, Muscle5, and HMMER;
8. Place query sequences using EPA-NG;
9. And compute and record the distance to the true placement.

To generate random damage parameters, we sampled each parameter from a uniform distribution with endpoints for each parameter listed in Suppl. Table 5. We performed placement with each of the randomly generated parameter sets, so that each of the prunings used the same sets of parameters. In total, we simulated 10 trees, performed 10 prunings, and generated 100 random parameter sets. This yielded $10,000$ independent tree, alignment and damage parameters which were then aligned with 5 methods and placed using EPA-NG.

When using the tools MAFFT, ClustalO and Muscle5, the damaged query sequences and the reference sequences were joined together into a single file, that is all damaged sequences and reference sequences were aligned together. In contrast, HMMER is able to perform alignment for each query sequence separately using a profile built from the reference sequences. As such, the reference sequences and the damaged query sequences were given to HMMER separately. Additionally, HMMER does not output its alignment as a FASTA file, therefore we perform an additional conversion step from PSIBLAST (our chosen output for HMMER) to FASTA. The

Tool	Command
MAFFT	`mafft --quiet --auto <INPUT> > <OUTPUT>`
ClustalO	`clustalo --in <INPUT> --output <OUTPUT>`
Muscle5	`muscle -super5 <INPUT> -output <OUTPUT>`
HMMER (Build)	`hmmbuild --cpu 1 --dna <OUTPUT> <INPUT>`
HMMER (Align)	`hmmalign --dna --outformat PSIBLAST`
	`-o <OUTPUT> --mapali <REFERENCES> <PROFILE> <QUERIES>`

Supplementary Table 4: Table of commands used for the alignment tools for the analysis in Section 4. Text in `<>` is filled in for the specific files used, I.E. `<INPUT>` is the file with both reference sequence and damaged query sequences, and `<OUTPUT>` is the destination alignment file.

Parameter	Min	Max
ν	0.00	0.30
λ	0.15	1.00
δ_{ss}	0.00	0.65
δ_{ds}	0.00	0.1

Supplementary Table 5: Table of parameters for the random sampling of damage parameters. Each parameter was sampled from the distribution $U(\text{Min}, \text{Max})$.

code to convert from PSIBLAST to FASTA has been adapted from code found in PEWO to perform the same task.

Using the default algorithm in Muscle5, some alignment tasks took excessively long (over 50 hours before the task was terminated). Therefore, In order to get results in a timely fashion, we elected to use the `-super5` algorithm to align sequences when using Muscle5.

The commands used for all of the alignment tools are listed in Suppl. Table 4.

4.1 Results

We report the overall performance over all parameters for each alignment tool as a boxplot in Suppl. Figure 4. Also, we summarize the data using both regression using a generalized additive model, and a linear regression, which we present in Suppl. Figure 3.

In Suppl. Tables 6 and 7 we present the scaled regression coefficients and R^2 for two models from Equations 1 and 2, respectively. Our experiment shows that the models are essentially equivalent in terms of goodness of fit (in terms of adjusted R^2: 0.08274 vs 0.08275). Additionally, the scaled parameter coefficients for ν between the two models are extremely close (2.875×10^{-1} vs 2.877×10^{-1}).

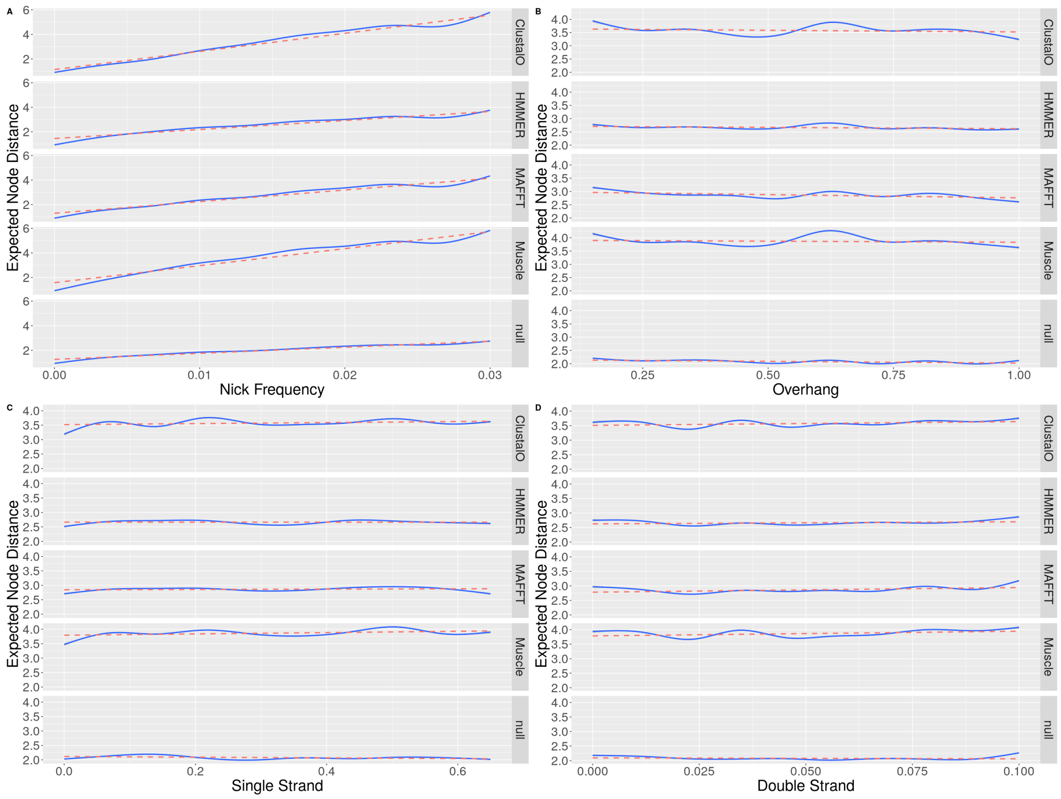

Supplementary Figure 3: Plot showing the expected node distance (eND) of EPA-NG when using the aligners Clustal Omega (ClustalO), HMMER, MAFFT, Muscle and the "null" alignment. The "null" alignment is a perfect alignment produced using information from the damage simulation. Blue solid line is the generalized additive model (GAM) regression as implemented in ggplot2. Red dashed line is the linear regression.

Similar to the results presented in the main text, the parameter with the largest impact is the ν. As in the main text, this indicates that the largest impact on placement accuracy when analyzing aDNA data is the read length. Also similar is the relative importance of each parameter. Specifically, after ν, δ_{ds} is the next most impactful parameter, followed by δ_{ss} and then finally λ (please see Suppl. Table 6).

An analogous result can be seen from the regression using Equation 2. The parameter coefficients in Suppl. Table 7 show that ν is the most impactful parameter. However, the joined terms $\lambda \times \delta_{ds}$ and $(1-\lambda) \times \delta_{ss}$ have about an impact that is about equivalent. Importantly, this shows that regardless of how we model the error, we find that ν, and consequently read length, is by far the most important parameter with regards to placement accuracy.

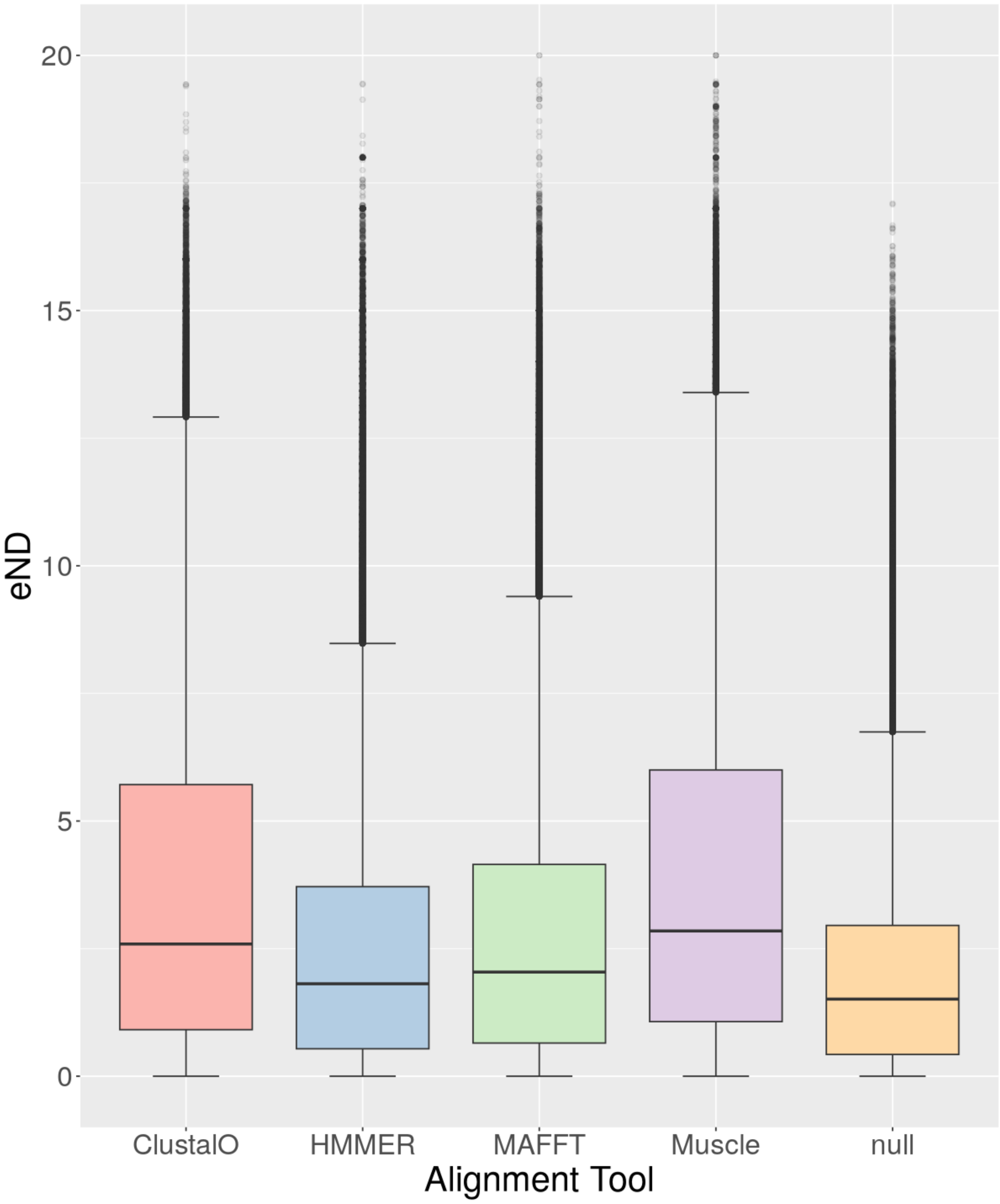

Supplementary Figure 4: Box plot of the overall performance of each alignment tool, as measured by expected node distance. Median eNDs for Clustal Omega, HMMER, MAFFT, Muscle, and “null” are 2.59, 1.81, 2.04, 2.85 and 1.51, respectively.

Parameter	Regression Coefficient
ν	$\mathbf{2.875 \times 10^{-1}}$
λ	-7.513×10^{-4}
δ_{ds}	$\mathbf{1.400 \times 10^{-2}}$
δ_{ss}	$\mathbf{8.183 \times 10^{-3}}$
Adjusted R^2	0.082 74

Supplementary Table 6: Table of scaled regression coefficients and R^2 for the model eND $\sim \nu + \lambda + \delta_{ds} + \delta_{ss}$. Bold indicates the value is significant at the $p < 0.01$ level.

Parameter	Regression Coefficient
ν	$\mathbf{2.877 \times 10^{-1}}$
$\lambda \times \delta_{ds}$	$\mathbf{1.695 \times 10^{-2}}$
$(1 - \lambda) \times \delta_{ss}$	$\mathbf{1.176 \times 10^{-2}}$
Adjusted R^2	0.082 75

Supplementary Table 7: Table of scaled regression coefficients and R^2 for the model eND $\sim \nu + \lambda \times \delta_{ds} + (1 - \lambda) \times \delta_{ss}$. Bold indicates that the value is significant at the $p < 0.01$ level.

5 Investigation of the Damage Parameter Space

As mentioned in the main text, a detailed exploration of all aDNA damage parameters would be computationally prohibitive. Therefore, we sought to constrain our detailed examinations to parameters which had a large impact. To this end, we performed an initial investigation on ds06 and evaluated a much larger grid of parameters. The parameters explored in this case are shown in Suppl. Table 9.

A plot of the regressions using the same parameters as the main paper using this detailed examination are presented in Suppl. Figure 5. Additionally the regression parameters from the models shown in Eqs. 1 and 2 are shown in Suppl. Table 10. As both the plots and the regression coefficients show, most important parameter by far is the nick frequency ν with regards to placement accuracy. With these results, we chose to restrict our search to a detailed examination of the effects of nick frequency for all datasets.

Dataset	Subplot	Software				
		APPLES	EPA-NG	`pplacer`	RAPPAS	All
ds01	**A**	**0.1053**	0.1417	0.1704	0.2180	0.1579
	**B**	**0.0175**	0.1404	0.1704	0.2180	0.1404
	**C**	**0.0702**	0.1079	0.1069	0.1028	0.0859
	**D**	**0.0702**	0.1038	0.1051	0.1028	0.0859
ds02	**A**	**0.0455**	0.0622	0.0659	0.0914	0.0617
	**B**	0.0455	**0.0414**	0.0739	0.0958	0.0568
	**C**	0.0455	**0.0051**	0.0183	0.0227	0.0240
	**D**	0.0455	**0.0050**	0.0190	0.0227	0.0229
ds03	**A**	0.2158	0.1926	**0.1763**	0.1776	0.1849
	**B**	0.1935	0.2186	**0.1806**	0.1864	0.1935
	**C**	0.1818	**0.0199**	0.0213	0.0421	0.0351
	**D**	0.1818	**0.0189**	0.0199	0.0323	0.0323
ds04	**A**	0.1579	0.1695	0.1805	**0.1148**	0.1601
	**B**	0.0385	0.1527	**0.1429**	0.1729	0.1479
	**C**	0.0370	**0.0000**	**0.0000**	0.0345	0.0000
	**D**	0.0370	**0.0000**	**0.0000**	**0.0000**	0.0000
ds05	**A**	0.0124	**0.0061**	0.0231	0.0186	0.0145
	**B**	**0.0119**	0.0145	0.0241	0.0248	0.0186
	**C**	0.0145	**0.0001**	0.0001	0.0040	0.0021
	**D**	0.0145	**0.0001**	0.0001	0.0040	0.0021
ds06	**A**	0.0080	**0.0056**	0.0088	0.0103	0.0080
	**B**	0.0081	**0.0062**	0.0080	0.0102	0.0081
	**C**	0.0051	0.0005	**0.0003**	0.0031	0.0018
	**D**	0.0054	0.0005	**0.0003**	0.0030	0.0014
ds07	**A**	0.0057	**0.0018**	0.0030	0.0068	0.0039
	**B**	0.0031	**0.0022**	0.0036	0.0088	0.0035
	**C**	0.0082	0.0007	**0.0004**	0.0006	0.0012
	**D**	0.0076	0.0007	**0.0002**	0.0003	0.0010
Best Count	**A**	2	3	1	1	
	**B**	2	3	2	0	
	**C**	1	4	3	0	
	**D**	1	4	3	1	

Supplementary Table 8: Table of median normalized eND for the histograms in Figures 3 and 4. Table is grouped by dataset, and subplot. Normalized eND for each software is computed using the parameters corresponding to the subplot in Figures 3 and 4. Specifically: **A** is restricted to $\nu = 0.025, \lambda = 0.15, \delta_{ss} = 0.65$, and $\delta_{ds} = 0.015$; **B** is restricted to $\nu = 0.025, \lambda = 1.0, \delta_{ss} = \delta_{ds} = 0.0$; **C** is restricted to $\nu = 0.0, \lambda = 0.15, \delta_{ss} = 0.65$, and $\delta_{ds} = 0.015$; and **D** is restricted to $\lambda = 1.0, \nu = \delta_{ss} = \delta_{ds} = 0.0$. For each row, the tool with the lowest normalize eND is shown in bold. In the case of a tie, multiple entries will be shown in bold. The final 4 rows counts the number of datasets and aDNA damage scenarios for which that tool was the best (or tied with the best).

Parameter	Values
ν	0.0000, 0.0001, 0.0005, 0.0010, 0.0050, 0.0100, 0.0200, 0.0250, 0.030, 0.050, 0.100
λ	0.01, 0.15, 0.30, 0.35, 0.40, 0.75, 1.00
δ_{ss}	0.00, 0.3, 0.6, 0.65, 0.7, 0.85, 1.0
δ_{ds}	0.000, 0.005, 0.010 0.015, 0.020, 0.100

Supplementary Table 9: Table containing the parameter values used in our experiments. End points for each parameter are based on the values estimated in Briggs et al. (2007).

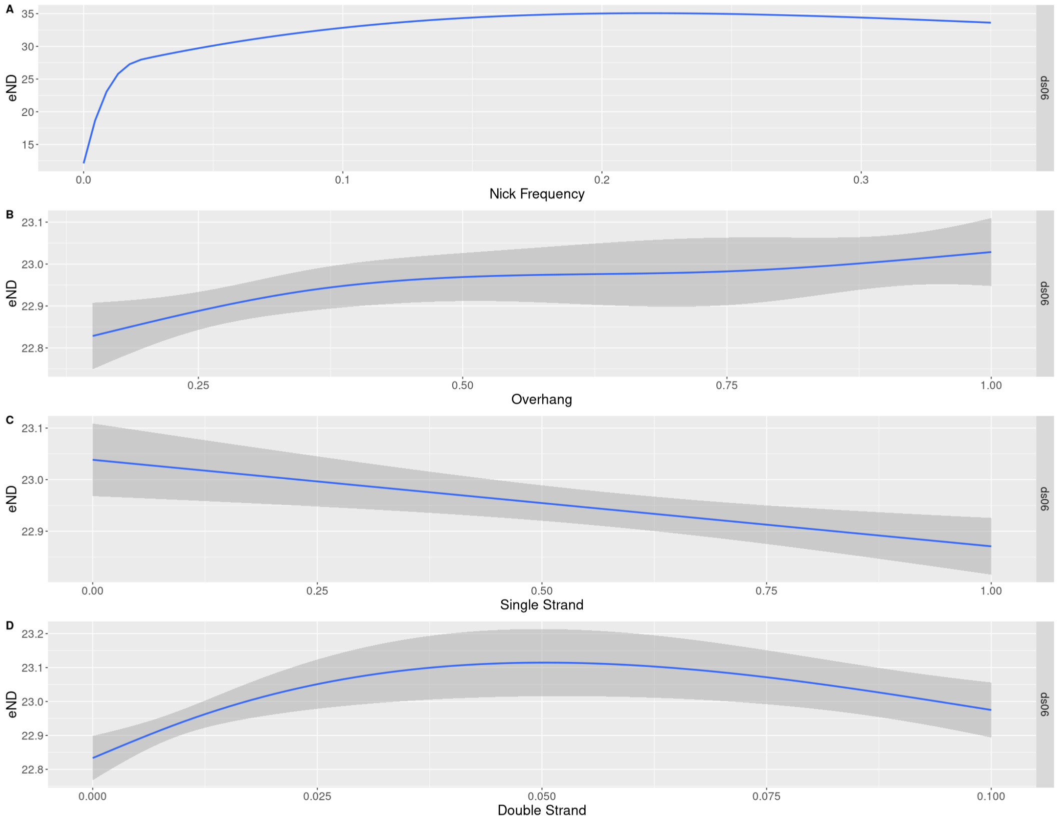

Supplementary Figure 5: Parameter regressions for ds06 with parameters as specified in Suppl. Table 9.

6 PEWO Prunings

In Suppl. Table 11 we present summary statistics for the pruning sizes for each dataset. Pruning sizes differ between datasets due to the fact that PEWO will prune entire subtrees, and so the realized size of a pruning is topology dependant.

For the most part, only a few taxa were pruned (the median for most datasets is between 1.0 and 2.5). However, the maximum size of each pruning was much larger, up to 84 for ds01. The wide range of pruning sizes indicates that PEWO generates placement problems that have a wide range in placement challenge, as increasing the number of taxa removed reduces the amount of data that is used to conduct the placement. Furthermore, this indicates that the results in the main paper represent a wide range of placement scenarios, and that the results are robust to different applications of phylogenetic placement.

Supplemental Material (PyGargammel Verfication)

The following is the output of a Jupyter notebook containing prose explaining the tests used to verify PyGargammel, and the code which performs the tests. The notebook itself is a part of the supplementary material contained in the Dryad repository specified the main text.

It is important to know that the database `sds.db` contains information from fragments generated in the assessment of the synthetic databases. Here, we use the synthetic datasets as they have a known and controlled length, which is a large factor in verifying the correctness of the output of PyGargammel.

```
import pandas
import numpy
import seaborn
import sqlite3

conn = sqlite3.connect('./sds.db')

nfs = [0.0, 0.0001, 0.0005, 0.001, 0.005, 0.01, 0.02, 0.0250, 0.03]
ovs = [0.15, 0.3, 0.35, 0.4, 0.75, 1.00]
sss = [0.0, 0.65]
dss = [0.0, 0.015, 0.1]
```

First, we are going to verify that the implementation of overhangs is correct. Per the Briggs model, overhangs are modeled as being extended iteratively, with probability of the extension process stopping of ν. Therefore, in order to verify that the overhangs are correct, its suffices to show that the overhang lengths are distributed according to Geom(ν).

To do this, we will select all the left and right overhangs for every fragement, including the overhang parameter. We will restrict this search to `nf == 0.0` in order to simply the tests later, as the length of the overhang is limited by the length of the fragment.

```
overhang_query = """
select
    overhang_left,
    overhang_right,
    ov
from fragment
inner join
    taxa on fragment.taxa_id == taxa.id
    inner join dataset on taxa.dataset_id == dataset.id
where dataset.nf == 0.0
"""

df = pandas.read_sql(overhang_query, conn)
```

Once we have the data, we need to do a bit more filtering. Some of the overhang parameters included in the database are old versions of pygargammel which allowed for overhangs on both sides of the fragment. This is no longer the case, and these runs are not included in the final paper, so we will exclude them from the statistical analysis.

Additionally, in order to make testing easier, we are going to put both the left and right overhangs into a single column called `overhang`.

```
df = df.loc[df.ov.isin(ovs)]
df['overhang'] = df['overhang_left'] + df['overhang_right']
df
```

```
       overhang_left  overhang_right   ov  overhang
```

```
0                 0              1  0.3          1
1                 2              0  0.3          2
2                 0              1  0.3          1
3                 0              0  0.3          0
4                 6              0  0.3          6
...             ...            ...  ...        ...
14251             1              0  0.4          1
14252             3              0  0.4          3
14253             0              0  0.4          0
14254             0              0  1.0          0
14255             0              0  1.0          0

[14256 rows x 4 columns]
```

We are going to use a Likelihood Ratio Test to determine if the simulation is correct. In short, the likelihood ratio between the MLE of the overhang parameter $\hat{nu}$ and the intended value ν should be very close to 1.0. Specifically, we are computing

$$LR(x) = \frac{L(\nu_0|x)}{L(\hat{\nu}|x)}$$

To do this, we will define two helper functions: `ov_llh` to compute $\log L(\nu|x)$ and `compute_ov_llh_ratio` compute $\hat{\nu}$ and to compute the final ratio.

```python
def ov_llh(k: int, nu: float):
    return numpy.log((1-nu)**k * nu)

def ov_mle(x: list):
    return 1 / (numpy.mean(x) + 1)

def compute_ov_llh_ratio(x: list, nu: float):
    nu_mle = ov_mle(x)
    llhs_mle = [ov_llh(k, nu_mle) for k in x]
    llhs_nu = [ov_llh(k, nu) for k in x]
    return (numpy.sum(llhs_nu) - numpy.sum(llhs_mle))
```

We then use these functions to compute the LRTs for each value of ν. Our null hypothesis is that ν is the true parameter, and we will reject with $\alpha = 0.05$.

```python
ov_lrts = pandas.DataFrame({
    'ov': ovs,
    'mle':[
        ov_mle(df.loc[df.ov == ov]['overhang'])
        for ov in ovs
    ],
    'lrt': [
        numpy.exp(
            compute_ov_llh_ratio(
                df.loc[df.ov == ov]['overhang'],
                ov
            )
        ) for ov in ovs
    ],
    'n': [len(df.loc[df.ov == ov]['overhang']) for ov in ovs],
})
```

```
ov_lrts['reject'] = ov_lrts['lrt'] < .1465
ov_lrts
```

```
     ov       mle       lrt     n  reject
0  0.15  0.150095  0.999443  2376   False
1  0.30  0.298305  0.946922  2376   False
2  0.35  0.352889  0.883971  2376   False
3  0.40  0.394161  0.651205  2376   False
4  0.75  0.778506  0.001122  2376    True
5  1.00  1.000000  1.000000  2376   False
```

As we have shown, we fail to reject for all parameter values. This indicates that the simulation of overhangs is correct.

We now turn our attention to the verification of the nick process. This process is paramerized by a Nick Frequency value, which is abbreviated as `nf`. Each base as a probability `nf` of experiencing a nick. This nick produces a new fragment, which has a length equal to the number of "failed" attempts at nicking. However, there are a few facts about the length:

- The minimum length of a fragment is 1;
- The length of the fragment is constrained by the source sequence; and
- The final fragment is not generated by the same process as the other fragments. Specifically, the final fragment is (by definition) constrained by the number of remaining bases. In particular, the final two facts means that the process is not truly geometric. However, we can verify that the process is correct nonetheless.

First, we need to query the data, and compute an adjusted length. We adjust the length by subtracting 1 from the length, which will align the length with the functions to compute the MLE and LRT for the overhangs.

```
nf_query = """
select
    start,
    end,
    nf,
    dataset_name,
    direction,
    fragment.id as fid,
    dataset.id as did,
    taxa.id as tid,
    pruning,
    filtered
from fragment
inner join
    taxa,
    dataset
on fragment.taxa_id == taxa.id and
    taxa.dataset_id == dataset.id
"""

nf_df = pandas.read_sql(nf_query, conn)
nf_df['length'] = nf_df['end'] - nf_df['start']
nf_df['adj_length'] = nf_df['length'] - 1

nf_df
```

```
         start   end       nf  dataset_name    direction       fid     did  \
```

```
0              0  5003  0.0000        sds01  THREE_PRIME        1      1
1              0  5003  0.0000        sds01   FIVE_PRIME        2      1
2              0  5003  0.0000        sds01  THREE_PRIME        3      1
3              0  5003  0.0000        sds01   FIVE_PRIME        4      1
4              0  5003  0.0000        sds01   FIVE_PRIME        5      1
...          ...   ...     ...          ...          ...      ...    ...
7761114   4957  4970  0.0200        sds09  THREE_PRIME  7761115  32399
7761115   4970  5000  0.0200        sds09  THREE_PRIME  7761116  32399
7761116      0  5000  0.0005        sds09  THREE_PRIME  7761117  32400
7761117      0  1251  0.0005        sds09  THREE_PRIME  7761118  32400
7761118   1251  5000  0.0005        sds09  THREE_PRIME  7761119  32400

            tid  pruning  filtered  length  adj_length
0             1        1         0    5003        5002
1             2        1         0    5003        5002
2             3        1         0    5003        5002
3             4        1         0    5003        5002
4             5        1         0    5003        5002
...         ...      ...       ...     ...         ...
7761114  142558        7         1      13          12
7761115  142558        7         0      30          29
7761116  142559        7         0    5000        4999
7761117  142560        7         0    1251        1250
7761118  142560        7         0    3749        3748

[7761119 rows x 12 columns]
```

With the data, we will now compute:

- the MLE as `mle`;
- the LRT (as with overhangs) as `lrt`;
- the number of fragments as `n`;
- the realized 99%-tile as `p99`;
- the expected 99%-tile ad `ep99`;
- the expected number of fragments per sequence as `e_frag_count`; and
- the rejection status as `reject`.

The remaining field, `last_frag_sampling_prob`, will be explained later.

```python
from scipy.stats import geom
from scipy.special import binom

selected_fragments = (nf_df.length > 0)

nf_lrts = pandas.DataFrame({
    'nf': nfs,
    'mle':[
        ov_mle(nf_df.loc[(nf_df.nf == nf) & selected_fragments]['adj_length'])
        for nf in nfs
    ],
    'lrt': [
        numpy.exp(
            compute_ov_llh_ratio(
                nf_df.loc[(nf_df.nf == nf) & selected_fragments]['adj_length'],
                nf
            )
```

```python
        ) for nf in nfs
    ],
    'n': [len(nf_df.loc[(nf_df.nf == nf) &
              selected_fragments]['adj_length']) for nf in nfs],
    'p99': [nf_df.loc[(nf_df.nf == nf) &
            selected_fragments]['adj_length'].quantile(.99) for nf in nfs],
    'ep99': [geom(nf, loc=-1).isf(.01) for nf in nfs],
    'e_frag_count': [5000 / geom(nf, loc=-1).expect()
                     if nf != 0.0 else float('nan') for nf in nfs],
})
nf_lrts['last_frag_sampling_prob_100'] = \
        numpy.clip(1 - binom(nf_lrts['e_frag_count']- 1, 100) /
                    binom(nf_lrts['e_frag_count'], 100), 0, 1)
nf_lrts['last_frag_sampling_prob_50'] = \
        numpy.clip(1 - binom(nf_lrts['e_frag_count']- 1, 50) /
                   binom(nf_lrts['e_frag_count'], 50), 0, 1)
nf_lrts['reject'] = nf_lrts['lrt'] < .1465
nf_lrts
```

```
/tmp/ipykernel_90743/51725531.py:2: RuntimeWarning: divide by zero encountered in log
  return numpy.log((1-nu)**k * nu)
/usr/lib/python3.12/site-packages/scipy/stats/_distn_infrastructure.py:573: RuntimeWarning: expect(): s
  return self.dist.expect(func, a, loc, lb, ub, conditional, **kwds)
```

```
       nf       mle           lrt        n      p99     ep99  e_frag_count  \
0  0.0000  0.000200  0.000000e+00    14256  5034.00      NaN           NaN
1  0.0001  0.000300  0.000000e+00    21466  5034.00  46049.0     13.820107
2  0.0005  0.000698  0.000000e+00    49892  5015.00   9208.0     13.459050
3  0.0010  0.001200  0.000000e+00    85715  3626.86   4602.0     13.947654
4  0.0050  0.005194  8.169255e-117  371051   881.00    918.0     30.354844
5  0.0100  0.010203  5.536899e-65   728873   450.00    458.0     59.315114
6  0.0200  0.020194  1.531639e-30  1442604   226.00    227.0    119.730651
7  0.0250  0.025198  1.147843e-25  1800056   180.00    181.0    150.072613
8  0.0300  0.030248  1.696284e-33  2160811   149.00    151.0    154.639175

   last_frag_sampling_prob_100  last_frag_sampling_prob_50  reject
0                          NaN                         NaN    True
1                     1.000000                    1.000000    True
2                     1.000000                    1.000000    True
3                     1.000000                    1.000000    True
4                     1.000000                    1.000000    True
5                     1.000000                    0.842955    True
6                     0.835208                    0.417604    True
7                     0.666344                    0.333172    True
8                     0.646667                    0.323333    True
```

We can see that we reject the LRT for all `nfs`. However, we expect this to be true, as we have shown earlier, that the generating process is indeed *not* geometric. We can still verify however that we are generating the correct distribution. The `last_frag_sampling_prob` computes the probability of sampling the final fragment, which is not generated with a geometric process, if we sample 100 fragments after applying nicks for a sequence. That is, the probabiltiy that the final results will contain a fragment generated under a different process.

Below is a simplified reimplementation of the nicking process in PyGargammel. We are going to simulate generating fragments from sequences of 5000 bases (which is approximatly the same size as the fragments used for the above analysis). We are going to run the process three times: once with all fragments, once

removing only the last fragment, and once with sampling 10,000 fragments.

```
def produce_nicks(p, m):
    total = m
    nicks = []
    RNG = numpy.random.default_rng()
    while total > 0:
        n = RNG.geometric(p)
        nicks.append(numpy.clip(n, 0, total))
        total -= n
    return [n - 1 for n in nicks]

def produce_fragments(test_nf, n, k, remove_last):
    if test_nf == 0.0:
        return float('nan')
    sample = []

    while len(sample) < n:
        nicks = produce_nicks(test_nf, 5000)
        if remove_last:
            sample.extend(nicks[:-1])
        else:
            sample.extend(nicks)


    if k is not None and k < n:
        sample = numpy.random.choice(sample, k, replace=False)

    return numpy.exp(compute_ov_llh_ratio(sample, test_nf))

tmp_df = pandas.DataFrame({
    'nf': nfs,
    'n': [len(nf_df.loc[(nf_df.nf == nf)]['adj_length']) for nf in nfs],
})
tmp_df['expected_lrt_all'] = [
    produce_fragments(nf, n, None, False) for nf, n in zip(tmp_df.nf, tmp_df.n)
]
tmp_df['expected_lrt_remove_1'] = [
    produce_fragments(nf, n, None, True) for nf, n in zip(tmp_df.nf, tmp_df.n)
]
tmp_df['expected_lrt_sample'] = [
    produce_fragments(nf, n, 10000, False) for nf, n in zip(tmp_df.nf, tmp_df.n)
]
tmp_df['reject_all'] = (tmp_df['expected_lrt_all'] < .1465)
tmp_df['reject_remove_1'] = tmp_df['expected_lrt_remove_1'] < .1465
tmp_df['reject_sample'] = tmp_df['expected_lrt_sample'] < .1465
tmp_df
```

```
       nf       n  expected_lrt_all  expected_lrt_remove_1  \
0  0.0000   14256               NaN                    NaN
1  0.0001   21467      0.000000e+00           0.000000e+00
2  0.0005   49897      0.000000e+00           0.000000e+00
3  0.0010   85716      0.000000e+00           0.000000e+00
4  0.0050  371112     4.519998e-133          2.557450e-139
5  0.0100  728976      2.945541e-64           3.053580e-65
```

```
6  0.0200  1442811      3.741865e-34          3.014765e-32
7  0.0250  1800316      7.550612e-28          2.939754e-21
8  0.0300  2161165      1.620410e-23          4.046330e-21

   expected_lrt_sample  reject_all  reject_remove_1  reject_sample
0                  NaN       False            False          False
1         0.000000e+00        True             True           True
2        2.662739e-217        True             True           True
3         5.704957e-70        True             True           True
4         3.245815e-05        True             True           True
5         3.219904e-02        True             True           True
6         9.659516e-01        True             True          False
7         5.017264e-01        True             True          False
8         6.841110e-01        True             True          False
```

We see that the test fails if we include all the fragments generated or if we remove the final fragment. The second failure might be surprising, but there is a reason. While the last fragment is constrained by the length of the origin sequence directly, the length of the second to last fragment is also constrained by the length of the origin sequence, as in order for it to be the second to last fragment, it had to be short enough that there was room for another fragment. This argument continues, though the magnitude of the effect is reduced, to the third to last fragment, the fourth to last fragment, and so on.

We can get around this by subsampling the produced fragments. In this case, the test is still sensitive enough to reject in the case of a non-geometric process. That is, if the process was incorrect, it would be very unlikely to obtain a sample of 10,000 fragments which would fail to reject with an LRT.

```python
nf_lrts['sample_lrt'] = [
    numpy.exp(
        compute_ov_llh_ratio(
            numpy.random.choice(
                nf_df.loc[(nf_df.nf == nf) & selected_fragments].adj_length,
                10000),
            nf)
    ) for nf in nfs
]
nf_lrts['sample_reject'] = nf_lrts['sample_lrt'] < .1465
nf_lrts
```

```
/tmp/ipykernel_90743/51725531.py:2: RuntimeWarning: divide by zero encountered in log
  return numpy.log((1-nu)**k * nu)
```

```
       nf       mle           lrt        n      p99     ep99  e_frag_count  \
0  0.0000  0.000200  0.000000e+00    14256  5034.00      NaN           NaN
1  0.0001  0.000300  0.000000e+00    21466  5034.00  46049.0     13.820107
2  0.0005  0.000698  0.000000e+00    49892  5015.00   9208.0     13.459050
3  0.0010  0.001200  0.000000e+00    85715  3626.86   4602.0     13.947654
4  0.0050  0.005194  8.169255e-117   371051   881.00    918.0     30.354844
5  0.0100  0.010203  5.536899e-65   728873   450.00    458.0     59.315114
6  0.0200  0.020194  1.531639e-30  1442604   226.00    227.0    119.730651
7  0.0250  0.025198  1.147843e-25  1800056   180.00    181.0    150.072613
8  0.0300  0.030248  1.696284e-33  2160811   149.00    151.0    154.639175

   last_frag_sampling_prob_100  last_frag_sampling_prob_50  reject  \
0                          NaN                         NaN    True
1                     1.000000                    1.000000    True
2                     1.000000                    1.000000    True
```

```
3                        1.000000                        1.000000    True
4                        1.000000                        1.000000    True
5                        1.000000                        0.842955    True
6                        0.835208                        0.417604    True
7                        0.666344                        0.333172    True
8                        0.646667                        0.323333    True

      sample_lrt  sample_reject
0   0.000000e+00           True
1   0.000000e+00           True
2  1.789075e-221           True
3   5.903238e-70           True
4   4.026758e-07           True
5   2.552294e-01          False
6   5.177060e-01          False
7   9.532105e-01          False
8   9.961440e-01          False
```

And here we have the result that matches simulations, indicating that we are producing fragments correctly.

Finally, we turn our attention to the site damage process. There are two types of damage "C->T" and "G->A". Our goal is to assess the following criteria:

- The damage follows the expected distribution, derived from empirical data; and
- The amount of C->T and G->A damage is roughly equivalent, given the GC content of the sequence.

As usual, we start by querying for the data from the database.

```
damage_query = """
select
    fragment.id as fragment_id,
    damage.id as damage_id,
    dataset.id as dataset_id,
    taxa.id as taxa_id,
    dataset_name,
    damage_type,
    position as relative_position,
    ov,
    ds,
    nf,
    ss,
    start as fragment_start,
    end as fragment_end,
    overhang_left,
    overhang_right,
    fragment.direction as direction,
    pruning
from fragment
left join damage
    on fragment.id == damage.fragment_id
inner join taxa, dataset
    on taxa.id == fragment.taxa_id
    and dataset.id == taxa.dataset_id
where
    dataset_name == 'sds05'
"""
```

```
damage_df = pandas.read_sql(damage_query, conn, dtype = {
    'damage_id' : "Int64",
    'relative_position' : "Int64"
})
damage_df
```

```
         fragment_id  damage_id  dataset_id  taxa_id dataset_name damage_type  \
0            4371855    4401187       14401    80281       sds05        A->G
1            4371855    4401188       14401    80281       sds05        A->G
2            4371855    4401189       14401    80281       sds05        A->G
3            4371855    4401190       14401    80281       sds05        A->G
4            4371855    4401191       14401    80281       sds05        A->G
...              ...        ...         ...      ...          ...         ...
2951238      6117642    6158648       18000   112320       sds05        C->T
2951239      6117642    6158649       18000   112320       sds05        C->T
2951240      6117642    6158650       18000   112320       sds05        C->T
2951241      6117642    6158651       18000   112320       sds05        C->T
2951242      6117642    6158652       18000   112320       sds05        C->T

         relative_position    ov     ds      nf    ss  fragment_start  \
0                      416  0.30  0.015  0.0000  0.65               0
1                     1215  0.30  0.015  0.0000  0.65               0
2                     1657  0.30  0.015  0.0000  0.65               0
3                     1739  0.30  0.015  0.0000  0.65               0
4                     1990  0.30  0.015  0.0000  0.65               0
...                    ...   ...    ...     ...   ...             ...
2951238                910  0.75  0.100  0.0005  0.00            3855
2951239                983  0.75  0.100  0.0005  0.00            3855
2951240               1033  0.75  0.100  0.0005  0.00            3855
2951241               1062  0.75  0.100  0.0005  0.00            3855
2951242               1096  0.75  0.100  0.0005  0.00            3855

         fragment_end  overhang_left  overhang_right    direction  pruning
0                5003              0               4  THREE_PRIME        1
1                5003              0               4  THREE_PRIME        1
2                5003              0               4  THREE_PRIME        1
3                5003              0               4  THREE_PRIME        1
4                5003              0               4  THREE_PRIME        1
...               ...            ...             ...          ...      ...
2951238          5001              0               0   FIVE_PRIME        7
2951239          5001              0               0   FIVE_PRIME        7
2951240          5001              0               0   FIVE_PRIME        7
2951241          5001              0               0   FIVE_PRIME        7
2951242          5001              0               0   FIVE_PRIME        7

[2951243 rows x 17 columns]
```

Once we have the data, we need to compute some additional information to make the plot. In order to make the plot easier to generate, we are going to flip the position of `G->A` damage, so that position 0 is the end of the string.

```
damage_df['position'] = damage_df['relative_position'] + damage_df.fragment_start
damage_df['fragment_length'] = damage_df.fragment_end - damage_df.fragment_start
damage_df['backwards_position'] = damage_df.fragment_length - damage_df.relative_position - 1
```

```
damage_df['in_overhang'] = (
    (damage_df['relative_position'] < damage_df['overhang_left'])
                | (damage_df['backwards_position'] < damage_df['overhang_right'])
)
damage_df['plot_position'] = [
    (r[1]['relative_position']
     if r[1]['damage_type'] == 'C->T' else r[1]['backwards_position'])
    for r in damage_df.iterrows()
]
damage_df
```

```
         fragment_id  damage_id  dataset_id  taxa_id dataset_name damage_type  \
0            4371855    4401187       14401    80281        sds05        A->G
1            4371855    4401188       14401    80281        sds05        A->G
2            4371855    4401189       14401    80281        sds05        A->G
3            4371855    4401190       14401    80281        sds05        A->G
4            4371855    4401191       14401    80281        sds05        A->G
...              ...        ...         ...      ...          ...         ...
2951238      6117642    6158648       18000   112320        sds05        C->T
2951239      6117642    6158649       18000   112320        sds05        C->T
2951240      6117642    6158650       18000   112320        sds05        C->T
2951241      6117642    6158651       18000   112320        sds05        C->T
2951242      6117642    6158652       18000   112320        sds05        C->T

         relative_position    ov     ds      nf  ...  fragment_end  \
0                      416  0.30  0.015  0.0000  ...          5003
1                     1215  0.30  0.015  0.0000  ...          5003
2                     1657  0.30  0.015  0.0000  ...          5003
3                     1739  0.30  0.015  0.0000  ...          5003
4                     1990  0.30  0.015  0.0000  ...          5003
...                    ...   ...    ...     ...  ...           ...
2951238                910  0.75  0.100  0.0005  ...          5001
2951239                983  0.75  0.100  0.0005  ...          5001
2951240               1033  0.75  0.100  0.0005  ...          5001
2951241               1062  0.75  0.100  0.0005  ...          5001
2951242               1096  0.75  0.100  0.0005  ...          5001

         overhang_left  overhang_right    direction  pruning position  \
0                    0               4  THREE_PRIME        1      416
1                    0               4  THREE_PRIME        1     1215
2                    0               4  THREE_PRIME        1     1657
3                    0               4  THREE_PRIME        1     1739
4                    0               4  THREE_PRIME        1     1990
...                ...             ...          ...      ...      ...
2951238              0               0   FIVE_PRIME        7     4765
2951239              0               0   FIVE_PRIME        7     4838
2951240              0               0   FIVE_PRIME        7     4888
2951241              0               0   FIVE_PRIME        7     4917
2951242              0               0   FIVE_PRIME        7     4951

         fragment_length  backwards_position  in_overhang  plot_position
0                   5003                4586        False           4586
1                   5003                3787        False           3787
2                   5003                3345        False           3345
```

```
3                        5003             3263       False          3263
4                        5003             3012       False          3012
...                       ...              ...         ...           ...
2951238                  1146              235       False           910
2951239                  1146              162       False           983
2951240                  1146              112       False          1033
2951241                  1146               83       False          1062
2951242                  1146               49       False          1096

[2951243 rows x 22 columns]
```

And finally, we plot the damage positions and type. Here, we see the expected curve, where damage occurs near the ends of the read. Recall that this is the expected result from the so-called "smile curve" from Briggs (2007).

```
ss_df = damage_df.loc[
    (damage_df.in_overhang)
    &(damage_df.ov == 0.3)
    &(damage_df.ss == 0.65)
]

seaborn.histplot(ss_df,
                 x="plot_position",
                 stat='density',
                 binwidth=1,
                 hue="damage_type",
                 multiple='dodge')
```

```
<Axes: xlabel='plot_position', ylabel='Density'>
```

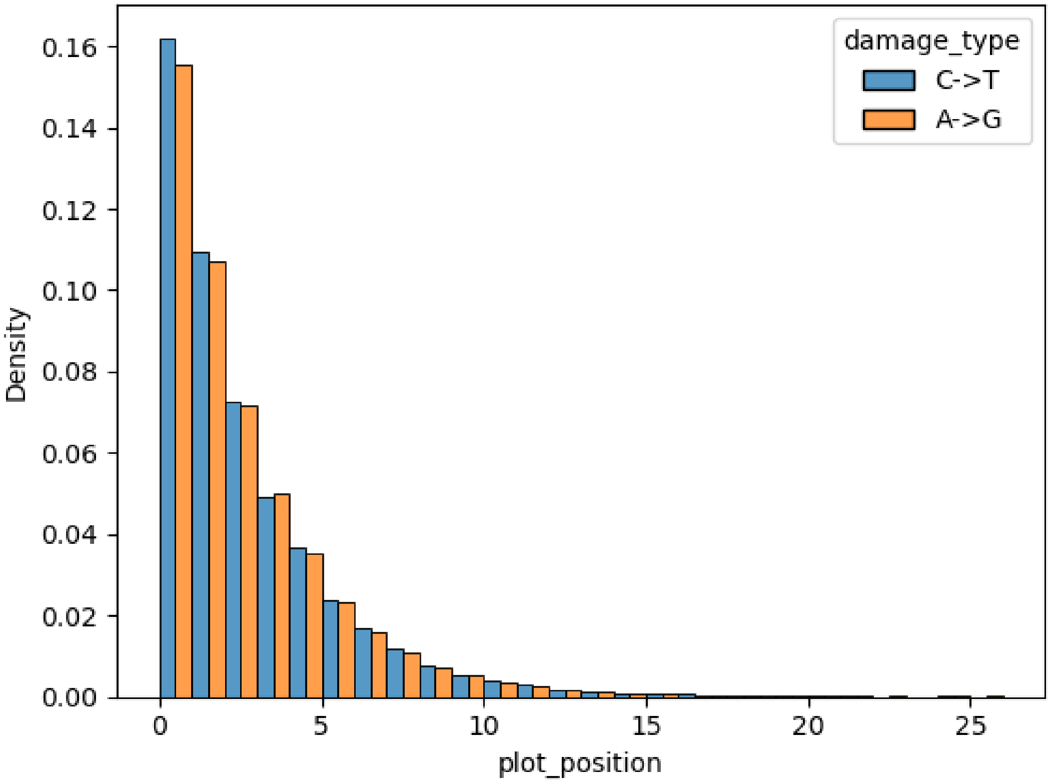

Model	Parameter	Regression Coefficient
Model 1	ν	$\mathbf{3.097 \times 10^{-1}}$
	λ	$\mathbf{3.621 \times 10^{-3}}$
	δ_{ds}	$\mathbf{-3.392 \times 10^{-3}}$
	δ_{ss}	1.735×10^{-3}
Adjusted R^2		0.095 96
Model 2	ν	$\mathbf{3.097 \times 10^{-1}}$
	$\lambda \times \delta_{ds}$	$\mathbf{-5.060 \times 10^{-3}}$
	$(1-\lambda) \times \delta_{ss}$	7.971×10^{-4}
Adjusted R^2		0.095 96

Supplementary Table 10: Table of scaled regression coefficients and R^2 for the models shown in Eqs. 1 and 2 for the detailed parameter exploration with ds06. Bold indicates the value is significant at the $p < 0.01$ level.

Dataset	Median	Mean	STD	Max
ds01	2.50	11.80	25.66	84.00
ds02	2.00	6.10	10.50	33.00
ds03	1.50	2.20	1.62	5.00
ds04	1.50	2.70	3.09	11.00
ds05	1.00	4.70	8.81	29.00
ds06	1.00	6.90	12.64	40.00
ds07	2.00	2.60	1.84	5.00

Supplementary Table 11: Table of summary statistics for the size of the prunings for each dataset. Pruning size is the number of taxa removed from the tree.

**Parameter**	**Values**
ν	0.0000, 0.0001, 0.0005, 0.0010, 0.0050, 0.0100, 0.0200, 0.0250, 0.030
λ	0.15, 0.30, 0.35, 0.40, 0.75, 1.00
δ_{ss}	0.00, 0.65
δ_{ds}	0.000, 0.015, 0.100

Supplementary Table 12: Table containing the parameter values used in our experiments. End points for each parameter are based on the values estimated in Briggs et al. (2007).

References

Adrian W. Briggs, Udo Stenzel, Philip L. F. Johnson, Richard E. Green, Janet Kelso, Kay Prüfer, Matthias Meyer, Johannes Krause, Michael T. Ronan, Michael Lachmann, and Svante Pääbo. Patterns of damage in genomic dna sequences from a neandertal. *Proceedings of the National Academy of Sciences*, 104:14616–14621, 9 2007. doi: 10.1073/pnas.0704665104. URL `http://dx.doi.org/10.1073/pnas.0704665104`.

Sean R. Eddy. Accelerated profile hmm searches. *PLoS Computational Biology*, 7:e1002195, 10 2011. doi: 10.1371/journal.pcbi.1002195. URL `http://dx.doi.org/10.1371/journal.pcbi.1002195`.

Robert C. Edgar. Muscle5: High-accuracy alignment ensembles enable unbiased assessments of sequence homology and phylogeny. *Nature Communications*, 13, 11 2022. doi: 10.1038/s41467-022-34630-w. URL `http://dx.doi.org/10.1038/s41467-022-34630-w`.

Kazutaka Katoh, Kazuharu Misawa, Kei-ichi Kuma, and Takashi Miyata. Mafft: a novel method for rapid multiple sequence alignment based on fast fourier transform. *Nucleic Acids Research*, 30(14):3059–3066, 07 2002. ISSN 0305-1048. doi: 10.1093/nar/gkf436. URL `https://doi.org/10.1093/nar/gkf436`.

Nhan Ly-Trong, Suha Naser-Khdour, Robert Lanfear, and Bui Quang Minh. Alisim: A fast and versatile phylogenetic sequence simulator for the genomic era. *Molecular Biology and Evolution*, 39, 2022. doi: 10.1093/molbev/msac092. URL `https://dx.doi.org/10.1093/molbev/msac092`.

Fabian Sievers, Andreas Wilm, David Dineen, Toby J Gibson, Kevin Karplus, Weizhong Li, Rodrigo Lopez, Hamish McWilliam, Michael Remmert, Johannes Söding, Julie D Thompson, and Desmond G Higgins. Fast, scalable generation of high-quality protein multiple sequence alignments using clustal omega. *Molecular Systems Biology*, 7, 1 2011. doi: 10.1038/msb.2011.75. URL `http://dx.doi.org/10.1038/msb.2011.75`.
